# Supplementary material for: CD4-binding site immunogens elicit heterologous anti-HIV-1 neutralizing antibodies in transgenic and wildtype animals
Source: Sci Immunol. Author manuscript; Available in PMC 2024 Feb 17. (PMC10202037; doi:10.1126/sciimmunol.ade6364)
Supplement: main supplementary [file NIHMS1887584-supplement-main_supplementary.pdf]

**Supplementary Materials for**

# CD4-binding site immunogens elicit heterologous anti-HIV-1 neutralizing antibodies in transgenic and wildtype animals

Harry B. Gristick and Harald Hartweger et al.

**Corresponding authors:**
Michel C. Nussenzweig nussen@rockefeller.edu
Pamela J. Bjorkman bjorkman@caltech.edu,

**The PDF file includes:**

Supplementary Materials and Methods

Figs. S1 to S8

Tables S1 to S8

## Supplementary Materials and Methods

### Generation of anti-idiotypic monoclonal antibodies

Mice were injected three times with purified IOMA iGL. 3 days after the final injection spleens were harvested and used to generate hybridomas at the Fred Hutchinson Antibody Technology Center. Hybridoma supernatants were initially screened against IOMA iGL to identify antigen-specific hybridomas. Supernatants from positive wells were then screened against a panel of monoclonal antibodies that included IOMA, IOMA iGL, and inferred germlines of other anti-HIV-1 antibodies that served as isotype controls using a high throughput bead array. We identified two hybridomas of interest; 3D3, which bound specifically to IOMA iGL, and 3D7, which bound to IOMA and IOMA iGL, which were subcloned from single cells. To produce recombinant anti-idiotypes, RNA was extracted from 1 × $10^6$ cells using the RNeasy kit (Qiagen), and the heavy and light chain sequences of the murine hybridomas were by obtained using the mouse Ig-primer set (69831; EMD Millipore) as described (*87*). Sequences were codon optimized, cloned into pTT3-based IgG expression vectors with human constant regions (*88*) using In-Fusion cloning (Clontech), expressed in 293 cells, and purified using Protein A chromatography.

### X-ray crystallography

Crystallization screens for IOMA iGL Fab were performed using the sitting drop vapor diffusion method at room temperature (RT) by mixing 0.2 µL Fabs with 0.2 µL of reservoir solution (Hampton Research) using a TTP Labtech Mosquito automatic microliter pipetting robot. IOMA iGL Fab crystals were obtained in 20% (v/v) PEG 2000, 0.1 M Sodium Acetate (pH 4.6). Crystals were looped and cryopreserved in reservoir solution supplemented with 20% glycerol and flash frozen in liquid nitrogen.

The crystal structure of IOMA iGL Fab was solved with data sets. A 1.9 Å-resolution structure of IOMA – 10-1074 – BG505 was solved with a single data set collected at 100 K and 1 Å resolution on Beamline 12-2 at the Stanford Synchrotron Radiation Lightsource (SSRL) with a Pilatus 6M pixel detector (Dectris) that was indexed and integrated with iMosflm v7.4, and then merged with AIMLESS in the CCP4 software package v7.1.018. The structure was determined by molecular replacement using Phaser with one copy of IOMA Fab (PDB 5T3Z). Coordinates were refined with PHENIX v1.19.2-4158 (*89*) with group B factor and TLS restraints. Manual rebuilding was performed iteratively with Coot v1.0.0 (*90*). Data refinement statistics are shown in Table S2, with > 98% of the residues in the favored region of the Ramachandran plot and < 1% in the disallowed regions.

## Cloning yeast libraries

Crystal structures of IOMA in complex with BG505 SOSIP.664 (PDB ID 5T3X and 5T3Z) were analyzed to determine mutations on gp120 that potentially could be beneficial for IOMA iGL binding. In addition, we modeled the crystal structure of IOMA iGL (PDB ID 7TQG) onto 426c.TM4ΔV1-3 (426c.TM4) gp120 (PDB ID 5FEC) and selected positions within gp120 that we predicted to be favorable for IOMA iGL binding. We chose 426c.TM4ΔV1-3 (426c TM4), an engineered clade C Env previously shown to activate B cell precursors of HIV-1 bNAbs targeting the CD4bs (*25*) as the starting point for our library design.

Yeast libraries were generated as described (*91*). Specifically, to generate the libraries of 426c gp120 variants we used degenerate oligos in conjunction with an overlap assembly polymerase chain reaction (PCR) method. Overlapping primers for the PCR assembly reactions were designed using Primerize (*92*) and shown in Table S6. NNK codons (where N = A/C/G/T and K = G/T) were utilized that encode for all 20 amino acids but decrease the chances of introducing a premature stop codon. Two different DNA fragments (426c library fragment 1 and 2) were synthesized first and then linearized in a final PCR step to generate the full-length 426c gp120 library used in yeast transformation. To obtain the full-length 426c gp120, a final PCR reaction was performed in which the PCR products of the 426c Library Fragment 1 and 2 were used as a template. Primers were used with overhangs complementary to the yeast display vector pCTCON-2 necessary for the homologous recombination in yeast. Library 2 was cloned in a similar manner as Library 1, but using a different set of primers as shown in Table S6 based on results from Library 1.

## Yeast transformation

The yeast display vector pCTCON-2 was used for cell surface display of the 426c gp120 proteins in *Saccharomyces cerevisiae* (*S. cerevisiae*) strain EBY100. A primary culture of 5 mL 2x YPD (40 g/L glucose, 20 g/L peptone, 20 g/L yeast extract) media was inoculated with a single *S. cerevisiae* EBY100 colony (freshly streaked on a YPD plate) and incubated overnight in a shaker at 30 °C and 250 rpm. 100 µL of the overnight yeast *S. cerevisiae* EBY100 cultures was transferred into 5 mL 2x YPD media and incubated overnight at 30 °C, 250 rpm. The following day, 300 mL 2x YPD media was inoculated with the overnight precultures to an $OD_{600}$ ~0.3 and was grown until an $OD_{600}$ ~1.6. 3 mL of sterile filtered Tris/DTT (0.462 g 1,4-dithiothreitol in 3 mL 1 M Tris, pH 8.0) and 15 mL sterile filtered 2 M LiAc/TE (1.98 g LiAc in 10 mL of TE (10 mM Tris, 1 mM EDTA) was added and the culture incubated for 15 min at 30 °C and 250 rpm. Yeast cells were then pelleted at 3,500 g for 3 min and washed with 50 mL ice-cold sterile filtered NewE buffer (0.6 g Tris base, 91.09 g Sorbitol (1 M), 73.50 mg $CaCl_2$ in

ddH$_2$O to a final volume of 500 mL, pH 7.5). After two additional wash steps, the pellet was re-suspended in 3 mL NewE buffer and 50 µg 426c library DNA insert and 10 µg pCTCON-2 vector (digested with NheI and BamHI) was added. 200 µL of this transformation mix was then aliquoted into pre-chilled 2 mm electroporation cuvettes (Bio-Rad) and electroporated at 1500 V with an average time constant of ~4.5 ms using a Gene Pulser Xcell Electroporation System (Bio-Rad), which was repeated for the entire transformation mix. After electroporation, yeast cells were directly recovered with 2 mL 2x YPD media and transferred into 50 mL cold 2x YPD media (final volume up to 200 mL 2x YPD media) and grown for 1 h at 30 °C and 250 rpm. Serial dilutions of the freshly transformed yeast culture were plated on SDCAA (20 g/L glucose, 6.7 g/L Difco yeast nitrogen base, 1.4 g/L Yeast Synthetic Drop-out Medium Supplements without histidine, leucine, tryptophan and uracil, 20 mg/L uracil, 50 mg/L histidine, 100 mg/L leucine) agarose plates to test the viability and size of the library. After 1 h, the culture was removed and the cells were pelleted and resuspended in 500 mL SDCAA media + carbenicillin (100 µg/mL final concentration) and grown for two days at 30 °C and 250 rpm. To confirm the genetic diversity of the library, a yeast colony PCR was performed on the liquid culture and the PCR product was sequenced. Sequencing reactions were performed at Laragen Inc (Culver City, CA). The sequence data was analyzed using SeqMan Pro (DNASTAR, v13.02). After two days, cells were pelleted and glycerol stocks were made by suspending ~$10^9$ yeast cells in 1 mL of freezing buffer (0.335 g Yeast Nitrogen Base, 1 mL glycerol in 50 mL H$_2$O, sterilized by filtration). Aliquots were flash frozen in liquid nitrogen and stored at -80 °C.

**Magnetic-activated cell sorting**

Magnetic-activated cell sorting (MACS) was used to remove transformants containing stop codons. After growing up the freshly transformed cells for two days in SDCAA, cells were pelleted and induced at an OD$_{600}$ ~1.0 in 100 mL SGCAA-carb (SDCAA prepared with 20 g/L galactose instead of glucose and supplemented with 100 µg/mL carbenicillin final concentration) for 20 h at 20 °C and 250 rpm. Yeast cells were washed 5 times with PBSF (PBS + 0.1% bovine serum albumin (BSA)) and $10^8$ cells were incubated with 400 µL PBSF and 100 µL µMACS™ anti-c-Myc MicroBeads (Miltenyi Biotec) for 45 min on a rotator at 4 °C. Cells were then pelleted and resuspended in 5 mL PBSF and sorted using a MidiMACS Separator magnet (Miltenyi Biotec) in combination with an LS column (Miltenyi Biotec) equilibrated in PBSF. Isolated cells were then grown for 2 days in 100 mL SDCAA-carb at 30 °C and 250 rpm and then induced again with SGCAA-carb for 20 h at 20 °C and 250 rpm.

**Yeast flow cytometry and cell sorting**

To prepare the yeast library for FACS analysis, cells were pelleted at 3000 rpm for 2 min and washed 5 times with PBSF. Cells were then stained at a density of $10^7$ cells/mL with 1:500 anti-c-Myc antibody conjugated to AlexaFluor488 (Abcam, ab190026) and 1 μM IOMA iGL and incubated for 1 – 2 h on a rotator at 4 °C. Cells were then washed twice with PBSF and resuspended in 200 μL PBSF with 1:1000 goat anti-human antibody conjugated to AlexaFluor647 (Abcam, ab190560, RRID:AB_2876372) and incubated for 30 min at 4 °C. Cells were then analyzed on a MACSQuant Analyzer (Miltenyi Biotec) or sorted using an SY3200 cell sorter system (Sony). In either case, non-transformed yeast cells and single-stained transformed samples stained with either anti-c-Myc or IOMA iGL IgG were used to set the gates for analysis and collection. Cells that stained double-positive for both c-Myc and IOMA iGL were collected and grown in 5 mL SDCAA-carb for 1 - 2 days at 30 °C and 250 rpm and then transferred to 100 mL SDCAA-carb for an additional 1 - 2 days at 30 °C and 250 rpm. Cells were then pelleted and resuspended in $H_2O$ and plated onto SDCAA-carb for 2 - 3 days at 30 °C. After multiple iterative rounds of sorting (three rounds for Library 1 and seven rounds for Library 2), sequences were recovered by colony PCR and sequence confirmed (Laragen). Primers were used with specific complementary regions to enable ligation of the linear product into the expression vector pTT5 using the Gibson assembly method for protein production. After construction, plasmids were isolated from *E.coli* using the QIAprep Miniprep kit (Qiagen) and confirmed by Sanger sequencing (Laragen).

**10x Genomics single cell processing and next generation V(D)J sequencing**

Cells were counted in the final injection volume, and 18,000 cells loaded onto a Chromium Controller (10x Genomics). Single-cell RNA-seq libraries were prepared using the Chromium Single Cell 5 v2 Reagent Kit (PN-1000265) according to manufacturer's protocol. Chromium Single Cell Mouse BCR Amplification Kit (PN-1000255) was used for VDJ cDNA amplification. After QC, 5' expression and VDJ Libraries were pooled 1:1 and sequenced on an Illumina NOVAseq S1 flowcell at the Rockefeller University Genomics Core.

**Computational Analyses of V(D)J sequences derived from IOMAgl mice by next generation sequencing**

The single-cell V(D)J assembly was carried out by Cell Ranger 6.0.1. A customized reference was created by adding the knocked-in IOMA iGL V(D)J genes to the mouse GRCm38 V(D)J reference so Cell Ranger could recognize and assemble the human/mouse chimera transcripts. Contigs associated with a valid cell barcode according to Cell Ranger were selected for downstream processing using seqtk version 1.3-r106 (https://github.com/lh3/seqtk).

IgBlast standalone version 1.14 (*101*) was used to annotate the immunoglobulin sequences based on a custom database with mouse and human V(D)J genes. Productive IG sequences with more than 20 reads of coverage and with any identified isotype were selected for downstream processing. Unexpectedly, although the IgBlast algorithm identified the V and J genes for 8010 LC sequences, it failed to annotate the CDR3, and consequently, the information regarding their functionality was missing. We extracted and submitted 7782 (97.15%) sequences corresponding to the knock-in LC to IMGT/V-QUEST (*102*), which successfully identified the CDR3 and provided the productivity information.

Cell barcodes associated with sequences coded by different V genes for either HC or LC were considered doublets and were subsequently removed from downstream analysis. HCs and LCs derived from the same cell were paired, and clones were assigned using our previously-described IgPipeline (*103, 104*) (https://github.com/stratust/igpipeline/tree/igpipeline2_timepoint_v2).

**Single cell antibody cloning**

The following modifications were applied to the described protocol from reference (*105*) Briefly, single cell RNA in 96-well plates was purified using magnetic beads (RNAClean XP, Beckman Coulter, Cat # A63987). RNA was eluted from the magnetic beads with 11 µL of a solution containing 14.5 ng/µL of random primers (Invitrogen, Cat # 48190011), 0.5% of Igepal Ca-630 (type NP-40, 10% in $dH_2O$, MP Biomedicals, Cat # 198596) and 0.6 U/µL of RNase inhibitor (Promega, Cat# N2615) in nuclease-free water (Qiagen, Cat # 129117), and incubated at 65 °C for 3 min. cDNA was synthesized by reverse transcription (SuperScript™ III Reverse Transcriptase 10,000 U, Invitrogen, Cat# 18080-044). cDNA was stored at −80 °C or used for antibody gene amplification by nested polymerase chain reaction (PCR) after addition of 10 µL of nuclease-free water.

Mouse antibody genes were amplified using HotstarTaq DNA polymerase (Qiagen Cat # 203209) with the primer sets specific for the $Igh^{IOMAiGL}$ and $Igk^{IOMAiGL}$ transgenes. Primer sequences and reaction mixes are provided in Table S8. Thermocycler conditions were as follows for annealing (°C)/elongation (s)/number of cycles: PCR1 (IgG, IgM and IgK): 51/55/50; PCR2 (IgG and IgM): 54/55/50; PCR2 (IgK): 50/55/50.

PCR products of antibody HC and LC genes were purified and Sanger-sequenced (Genewiz) and *ab1 files analyzed using our previously described IgPipeline (https://github.com/stratust/igpipeline/tree/igpipeline2_timepoint_v2) (*103, 104*). V(D)J sequences

were ordered as eBlocks (IDT) with short homologies for Gibson assembly and cloned into human IgG1 or human IgL2 expression vectors using the NEB Hifi DNA Assembly mix (NEB, Cat#E2621L). Plasmid sequences were verified by Sanger sequencing (Genewiz).

**SPR binding studies**

All SPR measurements were performed on a Biacore T200 (GE Healthcare) at 20 °C in HBS-EP+ (GE Healthcare) running buffer. IgGs were directly immobilized onto a CM5 chip (GE Healthcare) to ~3000 resonance units (RUs) using primary amine chemistry. A concentration series of monomeric gp120 core constructs (IGT2, IGT1, 426c TM4) were injected over the flow cells at increasing concentrations (top concentrations ranging from 600 μM to 10 μM) at a flow rate of 60 μL/min for 60 s and allowed to dissociate for 300 s. Regeneration of flow cells was achieved by injecting one pulse each of 10 mM glycine pH 2.0 at a flow rate of 90 μL/min. Kinetic analyses were used after subtraction of reference curves to derive on/off rates ($k_a$/$k_d$) and binding constants ($K_D$s) using a 1:1 binding model with or without bulk refractive index change (RI) correction as appropriate (Biacore T200 Evaluation software v3.0). Reported affinities represent the average of two independent experiments. SPR experiments that were not used to derive binding affinities or kinetic constants were done using a single high concentration (1 μM) to qualitatively determine binding versus no binding.

**Analysis Software**

Unless stated otherwise, Geneious Prime 2021.2.2, MacVector 18.2.0 and DNAStar SeqMan Pro 17.1.1 were used for sequence analysis and graphs were created using R language. Flow cytometry data were processed using Mac versions of FlowJo 10.7.2. and GraphPad Prism 9.3 and Microsoft Excel for Mac 16.54 were used for data analysis. Structural figures were made using PyMOL (Schrödinger, LLC) or ChimeraX (*109*). V(D)J gene assignments of NHP and murine antibodies were done using IMGT/V-QUEST (*102*). Sequence alignments were done using Clustal Omega (*110*).

# Figs. S1 to S8

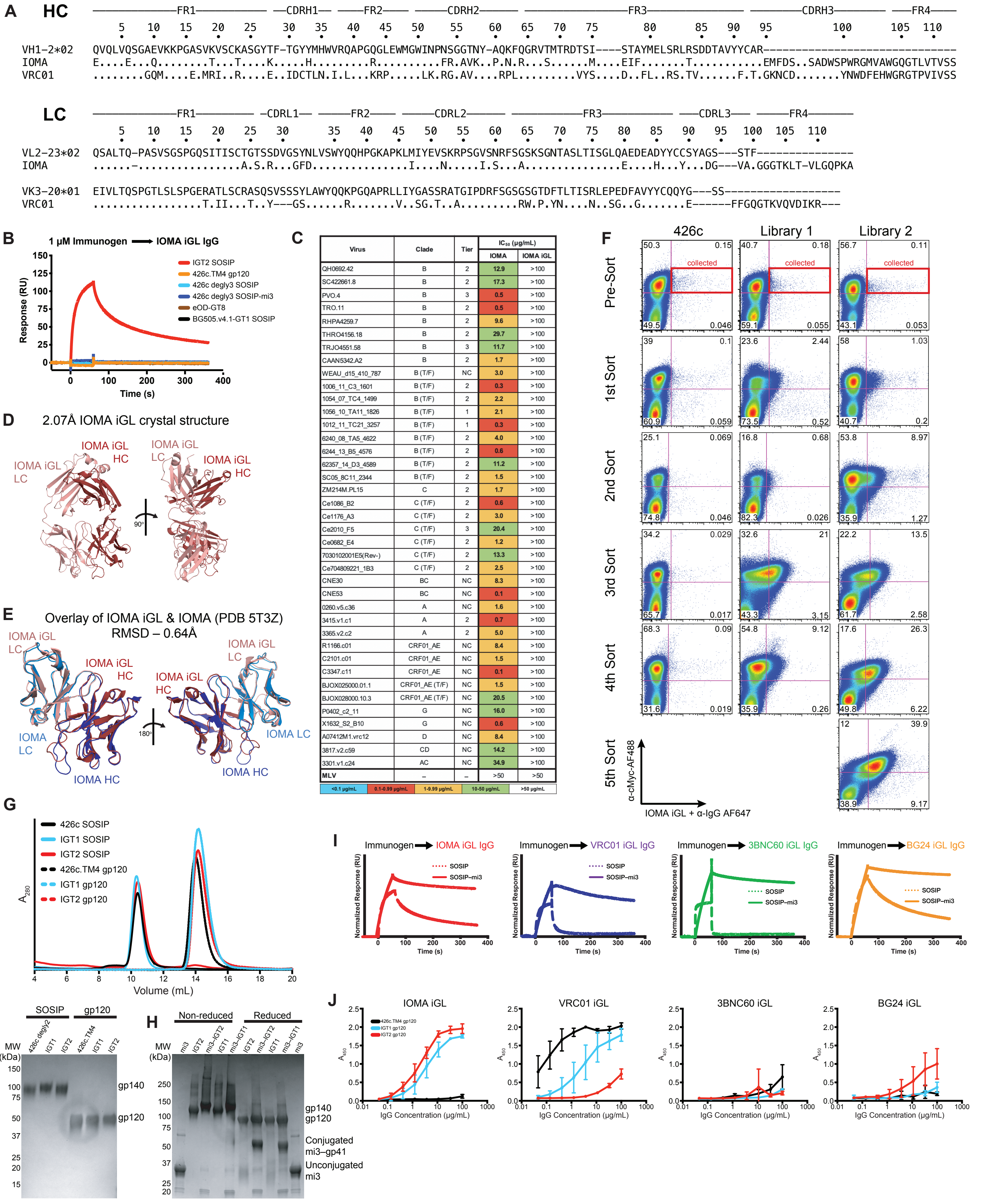


| Virus | Clade | Tier | IC50 (µg/mL) IOMA | IC50 (µg/mL) IOMA iGL |
|---|---|---|---|---|
| QH0692.42 | B | 2 | 12.9 | >100 |
| SC422661.8 | B | 2 | 17.3 | >100 |
| PVO.4 | B | 3 | 0.5 | >100 |
| TRO.11 | B | 2 | 0.5 | >100 |
| RHPA4259.7 | B | 2 | 9.6 | >100 |
| THRO4156.18 | B | 2 | 29.7 | >100 |
| TRJO4551.58 | B | 3 | 11.7 | >100 |
| CAAN5342.A2 | B | 2 | 1.7 | >100 |
| WEAU_d15_410_787 | B (T/F) | NC | 3.0 | >100 |
| 1006_11_C3_1601 | B (T/F) | 2 | 0.3 | >100 |
| 1054_07_TC4_1499 | B (T/F) | 2 | 2.2 | >100 |
| 1056_10_TA11_1826 | B (T/F) | 1 | 2.1 | >100 |
| 1012_11_TC21_3257 | B (T/F) | 1 | 0.3 | >100 |
| 6240_08_TA5_4622 | B (T/F) | 2 | 4.0 | >100 |
| 6244_13_B5_4576 | B (T/F) | 2 | 0.6 | >100 |
| 62357_14_D3_4589 | B (T/F) | 2 | 11.2 | >100 |
| SC05_8C11_2344 | B (T/F) | 2 | 1.5 | >100 |
| ZM214M.PL15 | C | 2 | 1.7 | >100 |
| Ce1086_B2 | C (T/F) | 2 | 0.6 | >100 |
| Ce1176_A3 | C (T/F) | 2 | 3.0 | >100 |
| Ce2010_F5 | C (T/F) | 3 | 20.4 | >100 |
| Ce0682_E4 | C (T/F) | 2 | 1.2 | >100 |
| 7030102001E5(Rev-) | C (T/F) | 2 | 13.3 | >100 |
| Ce704809221_1B3 | C (T/F) | 2 | 2.5 | >100 |
| CNE30 | BC | NC | 8.3 | >100 |
| CNE53 | BC | NC | 0.1 | >100 |
| 0260.v5.c36 | A | NC | 1.6 | >100 |
| 3415.v1.c1 | A | 2 | 0.7 | >100 |
| 3365.v2.c2 | A | 2 | 5.0 | >100 |
| R1166.c01 | CRF01_AE | NC | 8.4 | >100 |
| C2101.c01 | CRF01_AE | NC | 1.5 | >100 |
| C3347.c11 | CRF01_AE | NC | 0.1 | >100 |
| BJOX025000.01.1 | CRF01_AE (T/F) | NC | 1.5 | >100 |
| BJOX028000.10.3 | CRF01_AE (T/F) | NC | 20.5 | >100 |
| P0402_c2_11 | G | NC | 16.0 | >100 |
| X1632_S2_B10 | G | NC | 0.6 | >100 |
| A07412M1.vrc12 | D | NC | 8.4 | >100 |
| 3817.v2.c59 | CD | NC | 14.2 | >100 |
| 3301.v1.c24 | AC | NC | 34.9 | >100 |
| **MLV** | – | – | >50 | >50 |

<0.1 µg/mL | 0.1-0.99 µg/mL | 1-9.99 µg/mL | 10-50 µg/mL | >50 µg/mL

**Figure S1. Development and characterization of IGT1 and IGT2 immunogens. (A)** Amino acid alignment of IOMA and VRC01 to their respective germline V genes. **(B)** Representative SPR sensorgrams demonstrating no detectable binding of IOMA iGL to previously described immunogens (eOD-GT8, 426c.TM4, BG505.v4.1-GT1). This experiment was performed to qualitatively evaluate binding of IGT2 and previously described CD4bs immunogens to IOMA iGL rather than to derive affinity or kinetic constants. **(C)** Neutralization titers ($IC_{50}$s) of IOMA and IOMA iGL against a panel of 38 viruses and an MLV control. **(D)** 2.07 Å crystal structure of IOMA iGL Fab shown in two views. **(E)** Structural overlay of IOMA iGL Fab and IOMA Fab from BG505-bound structure (PDB 5T3Z).**(F)** Flow cytometric analysis of yeast cells expressing 426c.TM4 starting protein (left), Library 1 (middle), or Library 2 (right) stained with IOMA iGL IgG/anti-IgG AF647 (x-axis) and anti-cMyc AF488 (y-axis). **(G)** Representative size exclusion chromatography profiles and Coomassie-stained SDS-PAGE analysis for 426c.TM4 gp120, IGT1 gp120, and IGT2 gp120, 426c SOSIP, IGT1 SOSIP, and IGT2 SOSIP demonstrating that all of these proteins are monodispersed samples and that the selected mutations do not alter the stability or behavior of the immunogens compared to the starting proteins. **(H)** Coomassie-stained SDS–PAGE analysis for mi3, IGT2, IGT2-mi3, IGT1, and IGT1-mi3 under non-reducing and reducing conditions. **(I)** SPR sensorgrams demonstrating binding of IGT2 (dashed line) and IGT2-mi3 (solid line) to IOMA iGL IgG (red), VRC01 iGL IgG (purple), 3BNC60 iGL IgG (green), and BG24 iGL IgG (orange). IgG was immobilized to the CM5 chip and 1 µM SOSIP or 1 µM SOSIP-mi3 was flowed over the chip surface. **(J)** Representative ELISA binding curves measuring binding of 426c.TM4 gp120, IGT1 gp120, and IGT2 gp120 to the same iGL IgGs as in (I). Dots indicate mean and error bars indicate 95% confidence interval.

# Figure S2

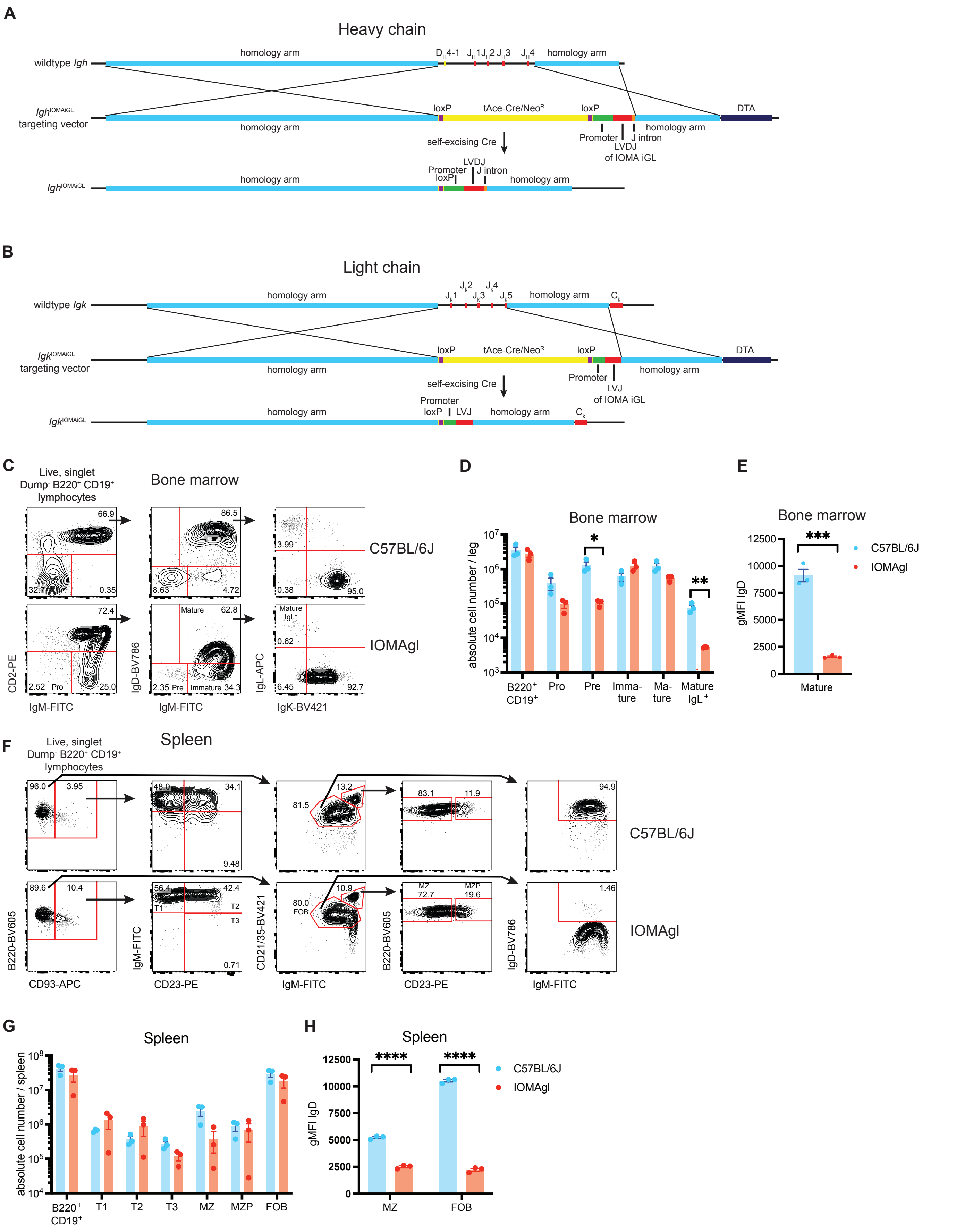
A
Heavy chain
wildtype Igh
homology arm
Igh IOMAiGL targeting vector
loxP
tAce-Cre/NeoR
DTA
Promoter
J intron
LVDJ
of IOMA iGL
self-excising Cre
Igh IOMAiGL
B
Light chain
wildtype Igk
Igk IOMAiGL targeting vector
LVJ
Igk IOMAiGL
C
Live, singlet
Dump- B220+ CD19+
lymphocytes
Bone marrow
C57BL/6J
IOMAgl
CD2-PE
IgM-FITC
IgD-BV786
IgL-APC
IgK-BV421
Pro
Pre
Immature
Mature
D
Bone marrow
absolute cell number / leg
B220+ CD19+
Pro
Pre
Imma-ture
Ma-ture
Mature IgL+
E
Bone marrow
gMFI IgD
Mature
F
Spleen
B220-BV605
CD93-APC
IgM-FITC
CD23-PE
CD21/35-BV421
IgD-BV786
T1
T2
T3
MZ
MZP
FOB
G
Spleen
absolute cell number / spleen
H
Spleen
gMFI IgD

**Figure S2. Targeting strategy and characterization of IOMAgl mice. (A)** In *Igh*$^{IOMAiGL}$ mice *Ighd4-1* to *Ighj4* are replaced by a self-excising Neomycin cassette followed by the mouse *Ighv9-4* promoter, a leader sequence (L) followed by the iGL version of the IOMA HC VDJ sequence and a *Ighj1* splice donor sequence. **(B)** In *Igk*$^{IOMAiGL}$ mice *Igkj1* to *Igkj5* are replaced by a self-excising Neomycin cassette followed by a mouse *Igkv3-12* promoter, a leader sequence followed by the iGL version of the IOMA lambda LC VDJ sequence and a *Igkj5* splice donor sequence. DTA, diphtheria toxin A **(C)** Flow cytometric analysis of B cell development in the bone marrow of control (C57BL/6J) or IOMAgl (*Igh*$^{IOMAiGL/IOMAiGL}$ *Igk*$^{IOMAiGL/IOMAiGL}$) mice. **(D)** Absolute cell number quantification from (C). **(E)** Geometric mean fluorescence intensity (gMFI) of IgD in mature recirculating B cells from the bone marrow. **(F)** Flow cytometric analysis of peripheral B cell development in the spleens of control (C57BL/6J) or IOMAgl mice. **(G)** Absolute cell number quantification from (F). **(H)** gMFI of IgD in marginal zone and follicular B cell. MZ, marginal zone B cells; MZP, marginal zone precursors; FOB, follicular B cells. Data from 1 of 2 independent experiments, each dot represents a data from 1 mouse. Bars represent mean ± SEM. Statistical analysis used unpaired t test.

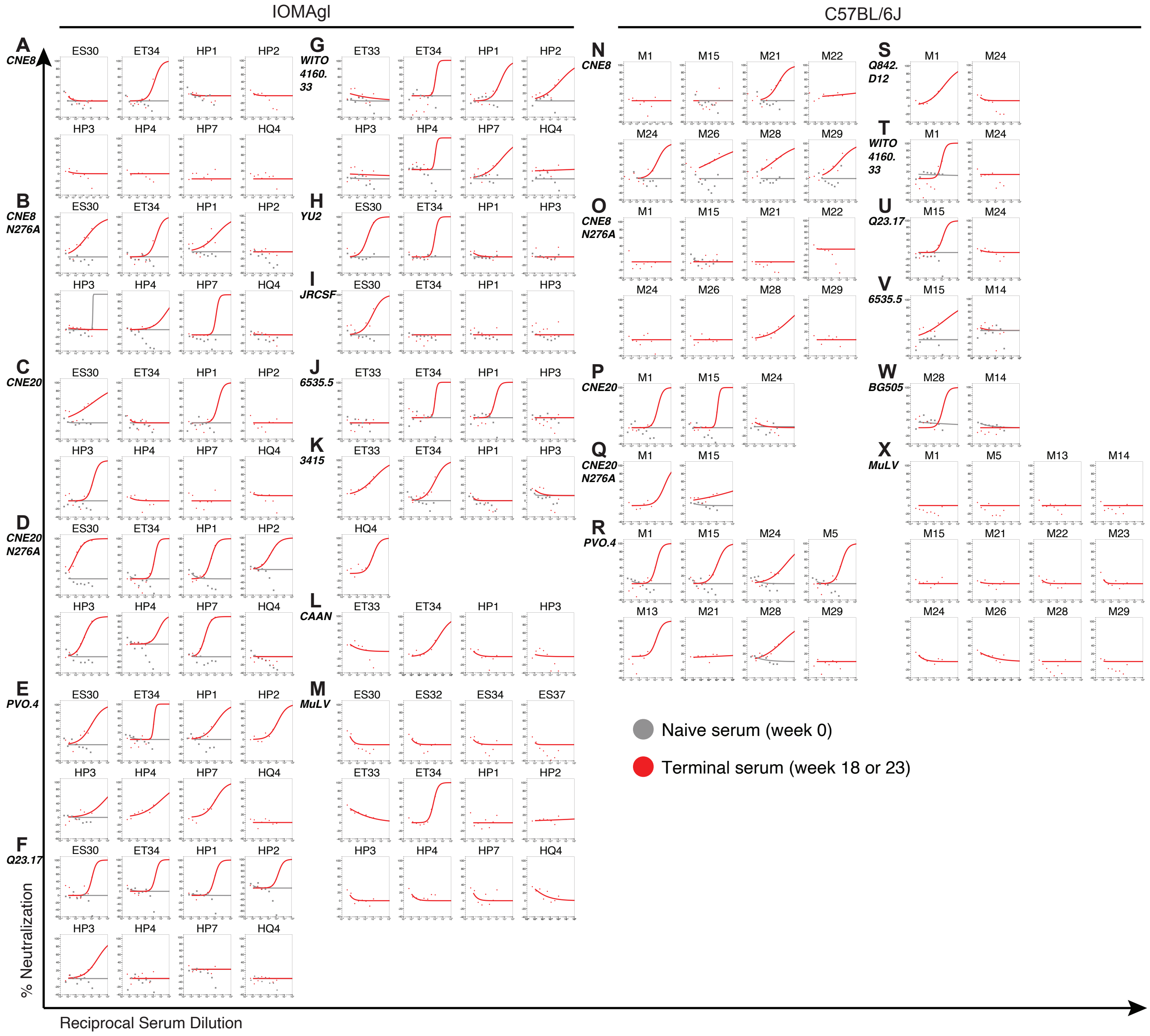


**Figure S3. Serum neutralization from immunized mice.** Neutralization curves of serum isolated from IOMA iGL transgenic mice (**A-M**) or C57BL/6J wildtype mice (**N-X**) against the following HIV strains or control MuLV: **(A,N)** CNE8, **(B,O)** CNE8 N276A, **(C,P)** CNE20, **(D,Q)** CNE20 N276A, **(E,R)** PVO.4, **(F,U)** Q23.17, **(G,T )** WITO4160.33, **(H)** YU2, **(I)** JRCSF, **(J, V)** 6535.5, **(K)** 3415_V1_C1, **(L)** CAAN5342.A2, **(M,X)** MuLV, (**S**) Q842.D12 and (**W**) BG505. Naïve serum was also tested against the same strains when available. Note that sera which showed neutralization activity of < 40% as listed in Table S3 are presented in Figure 2G as white rectangles; several of these sera neutralized strains above background including ET33 against PVO.4; ET34 against CNE20 N276A and Q23.17; HP1 against CNE8 N276A, CNE20, and WITO4160.33; HP2 against Q23.17; HP3 against Q23.17 and PVO.4; HP4 against CNE8 N276A, CNE20 N276A, and PVO.4.

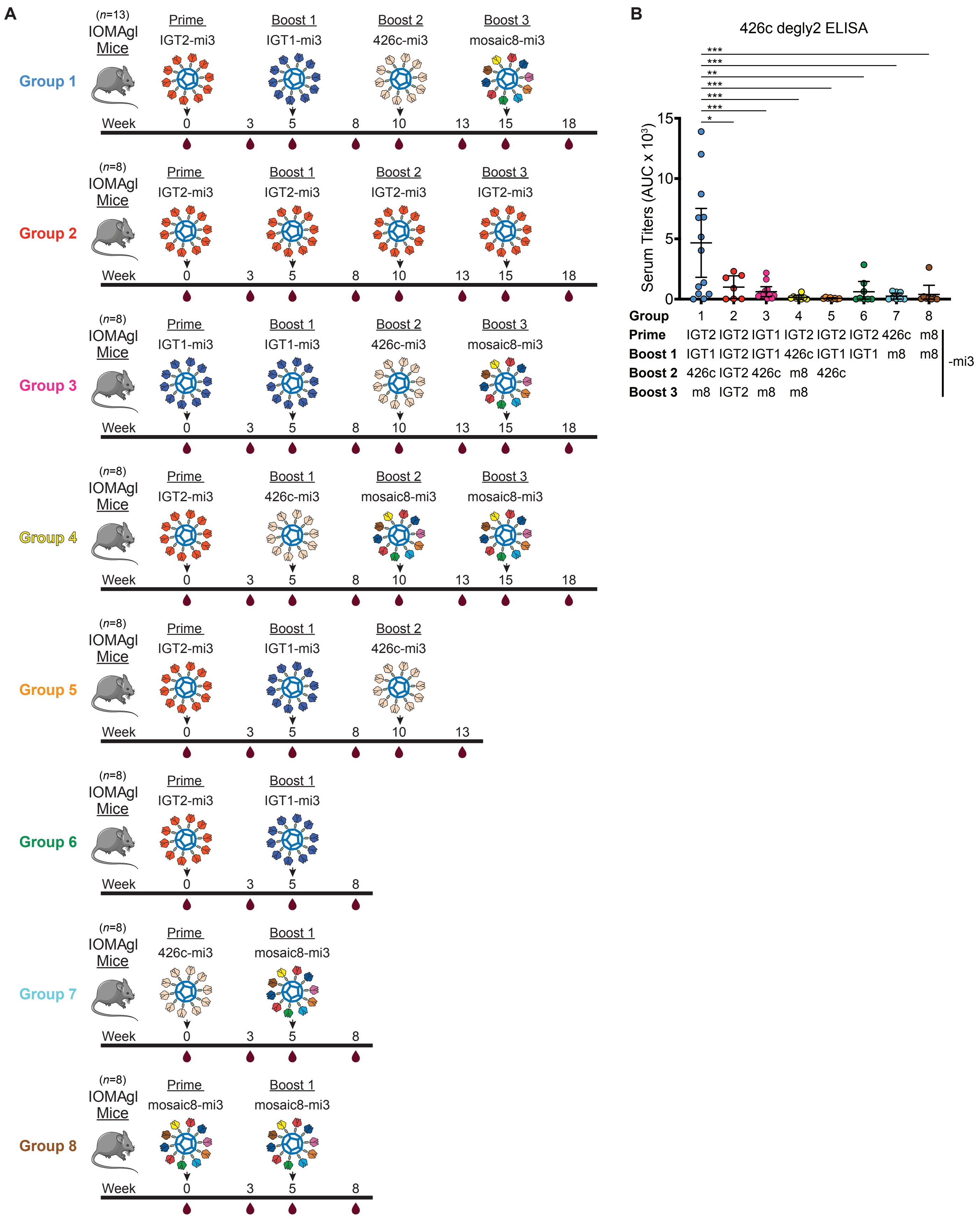
A
B
(n=13) IOMAgl Mice
Group 1
Prime IGT2-mi3
Boost 1 IGT1-mi3
Boost 2 426c-mi3
Boost 3 mosaic8-mi3
Week 0 3 5 8 10 13 15 18
(n=8) IOMAgl Mice
Group 2
Prime IGT2-mi3
Boost 1 IGT2-mi3
Boost 2 IGT2-mi3
Boost 3 IGT2-mi3
Week 0 3 5 8 10 13 15 18
(n=8) IOMAgl Mice
Group 3
Prime IGT1-mi3
Boost 1 IGT1-mi3
Boost 2 426c-mi3
Boost 3 mosaic8-mi3
Week 0 3 5 8 10 13 15 18
(n=8) IOMAgl Mice
Group 4
Prime IGT2-mi3
Boost 1 426c-mi3
Boost 2 mosaic8-mi3
Boost 3 mosaic8-mi3
Week 0 3 5 8 10 13 15 18
(n=8) IOMAgl Mice
Group 5
Prime IGT2-mi3
Boost 1 IGT1-mi3
Boost 2 426c-mi3
Week 0 3 5 8 10 13
(n=8) IOMAgl Mice
Group 6
Prime IGT2-mi3
Boost 1 IGT1-mi3
Week 0 3 5 8
(n=8) IOMAgl Mice
Group 7
Prime 426c-mi3
Boost 1 mosaic8-mi3
Week 0 3 5 8
(n=8) IOMAgl Mice
Group 8
Prime mosaic8-mi3
Boost 1 mosaic8-mi3
Week 0 3 5 8
426c degly2 ELISA
Serum Titers (AUC x 10$^3$)
0 5 10 15
*** *** ** *** *** *** *
Group 1 2 3 4 5 6 7 8
Prime IGT2 IGT2 IGT1 IGT2 IGT2 IGT2 426c m8
Boost 1 IGT1 IGT2 IGT1 426c IGT1 IGT1 m8 m8
Boost 2 426c IGT2 426c m8 426c
Boost 3 m8 IGT2 m8 m8
-mi3

**Figure S4. Screening immunization regimens to determine the optimal boosting strategy. (A)** Schematic and timeline of immunization strategies to determine the optimal regimen to elicit IOMA-like bNAbs. **(B)** Serum ELISA binding to 426c degly2 represented as AUC using serum samples isolated from mice at the end of the regimen. m8, mosaic8.

# Figure S5

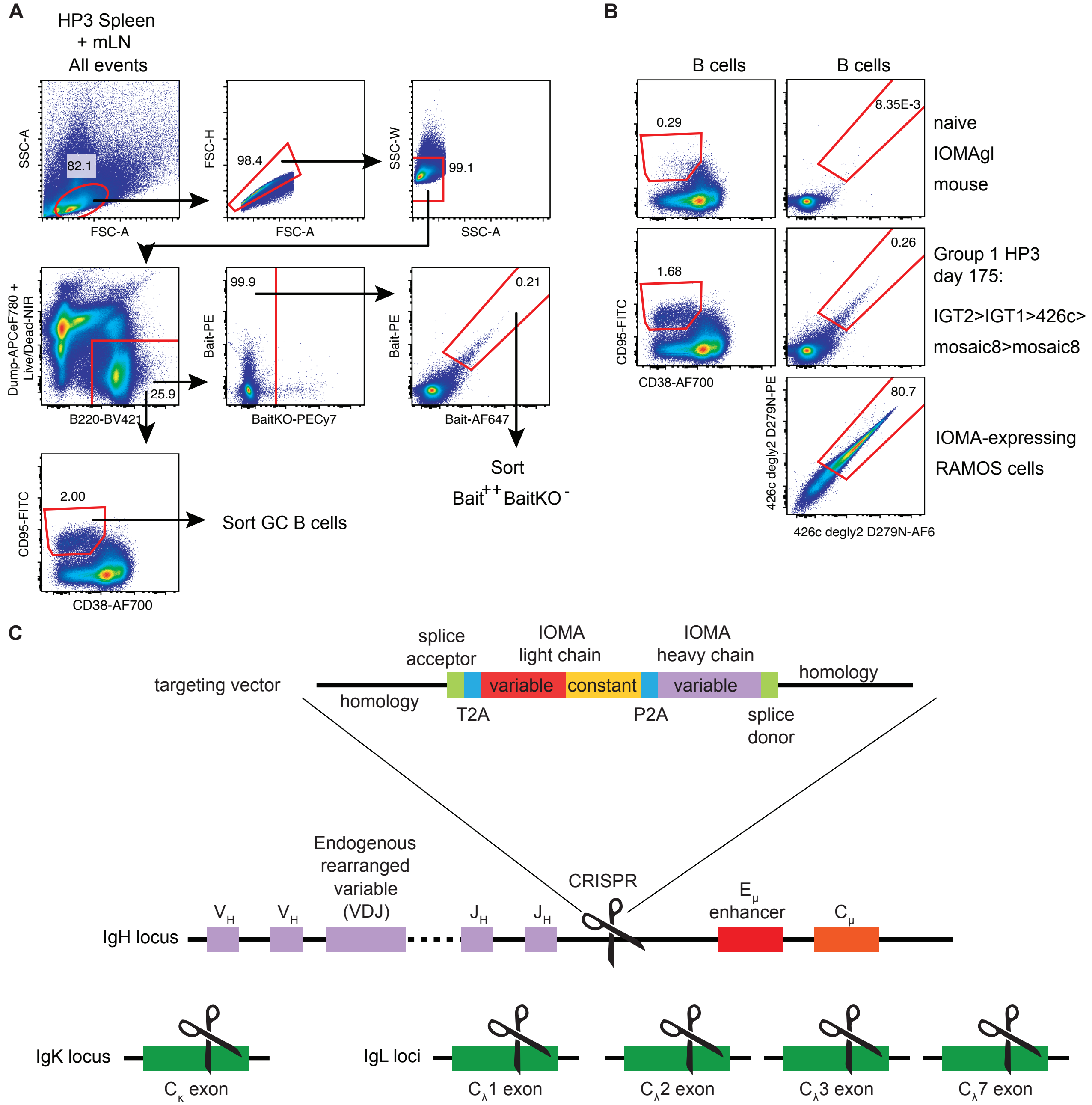


**Figure S5. Cell sorting strategies and sorting controls. (A)** Representative full gating of cell sorts for single cell Bait++ BaitKO- B cell cloning and 10x Genomics next generation VDJ sequencing of bulk-sorted GC B cells from splenic and mesenteric lymph nodes. Baits used were 426c degly2 D279N or CNE8 N276A with 426c degly2 D279N-CD4bs KO, the former is shown. **(B)** Induction of germinal center response and wt SOSIP-binding cells by immunization regimen (group 1). Naïve IOMAgl mouse splenocytes and IOMA-expressing RAMOS cells served as negative and positive control, respectively. (**C**) Gene editing strategy to generate IOMA-expressing RAMOS cells.

Simultaneous targeting of IgH, IgK and IgL loci with CRISPR/Cas9 to delete endogenous LCs and edit a promoterless tricistronic expression cassette into the IgH locus to express IOMA on the surface of RAMOS cells. A polycistronic mRNA was created using T2A and P2A sequences to induce ribosomal skipping (*96*).

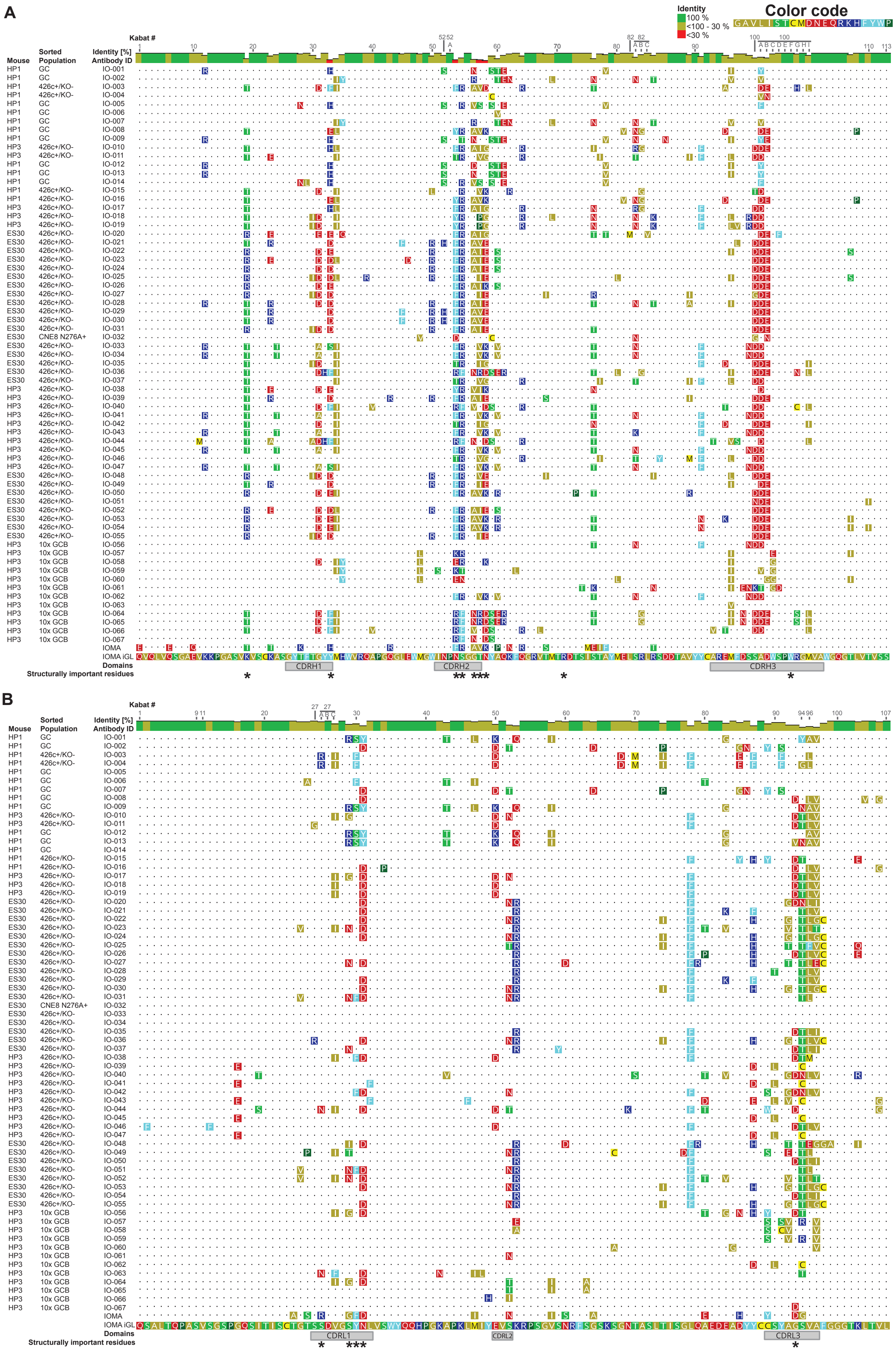
A
Color code
Identity
100 %
<100 - 30 %
<30 %
Kabat #
Mouse
Sorted Population
Identity [%]
Antibody ID
IOMA
IOMA iGL
Domains
CDRH1
CDRH2
CDRH3
Structurally important residues
B
CDRL1
CDRL2
CDRL3

**Figure S6. Amino acid alignments of selected IOMAgl mouse-derived antibodies. (A)** $V_H$ alignment of cloned antibodies IO-001 to IO-067 that were expressed and tested for Env binding. IOMA iGL and IOMA sequence at the bottom as reference. Mouse ID and population sorted are indicated. Differences to IOMA iGL are highlighted using chemically similar color coding; dots indicate identical residues to IOMA iGL. Kabat numbering and percent identity of residues are indicated on top. Domains and residues of structural importance are annotated below. **(B)** as above but corresponding $V_L$ alignment.

# Figure S7

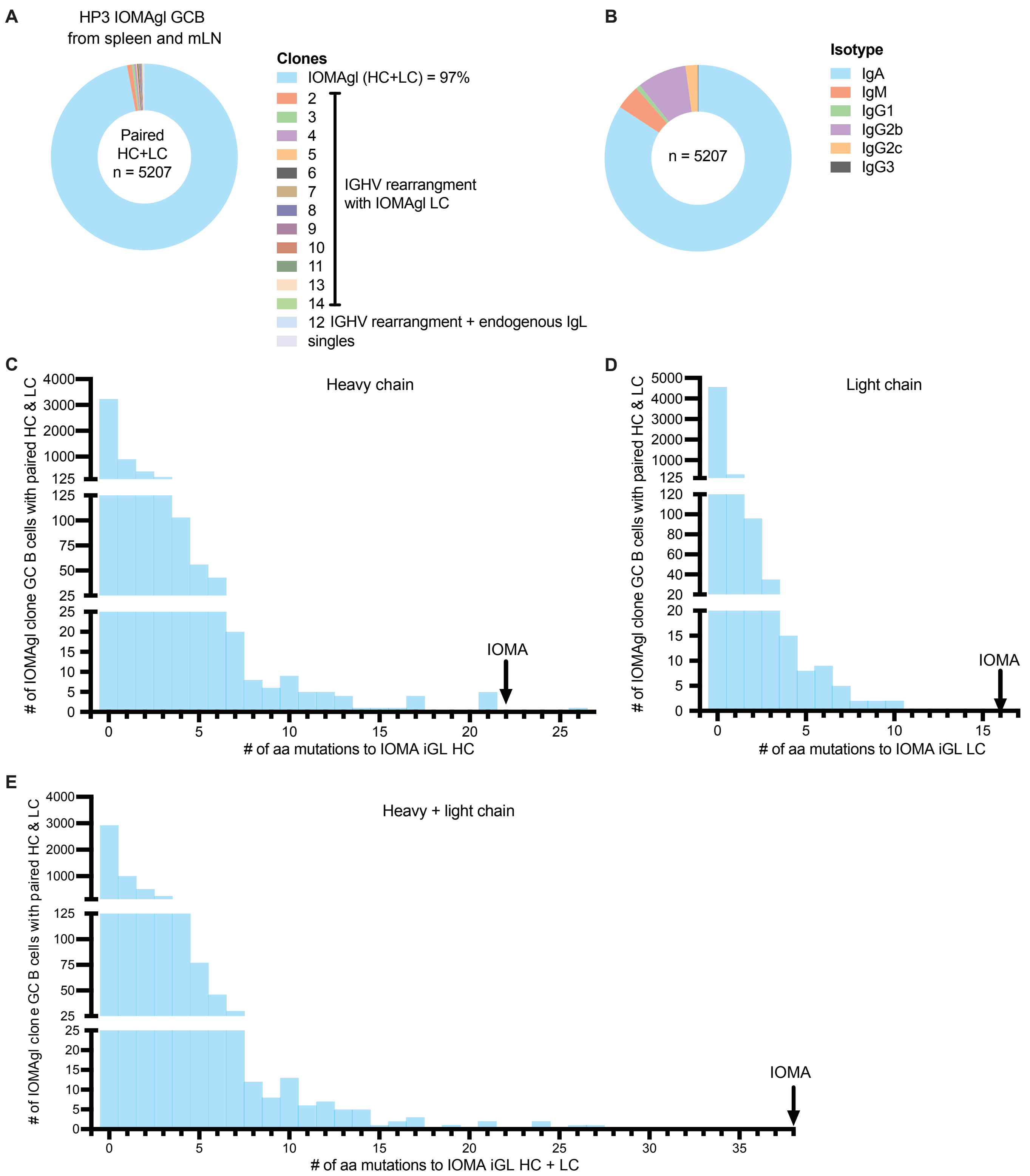


**Figure S7. Next generation single cell VDJ analysis determines the extent of mutations in germinal centers of IOMAgl mice. (A)** Clonal analysis of paired HC and LC sequences from splenic and mesenteric lymph node germinal center B cells of IOMAgl mouse HP3. **(B)** Isotype distribution among these cells. **(C)** Frequency distribution of the number of amino acid mutations to IOMA iGL in

the HC sequences of these cells. **(D)** Frequency distribution of the number of amino acid mutations to IOMA iGL in the LC sequences of these cells. **(E)** Frequency distribution of the number of amino acid mutations to IOMA iGL in the paired HC and LC sequences of these cells.

A

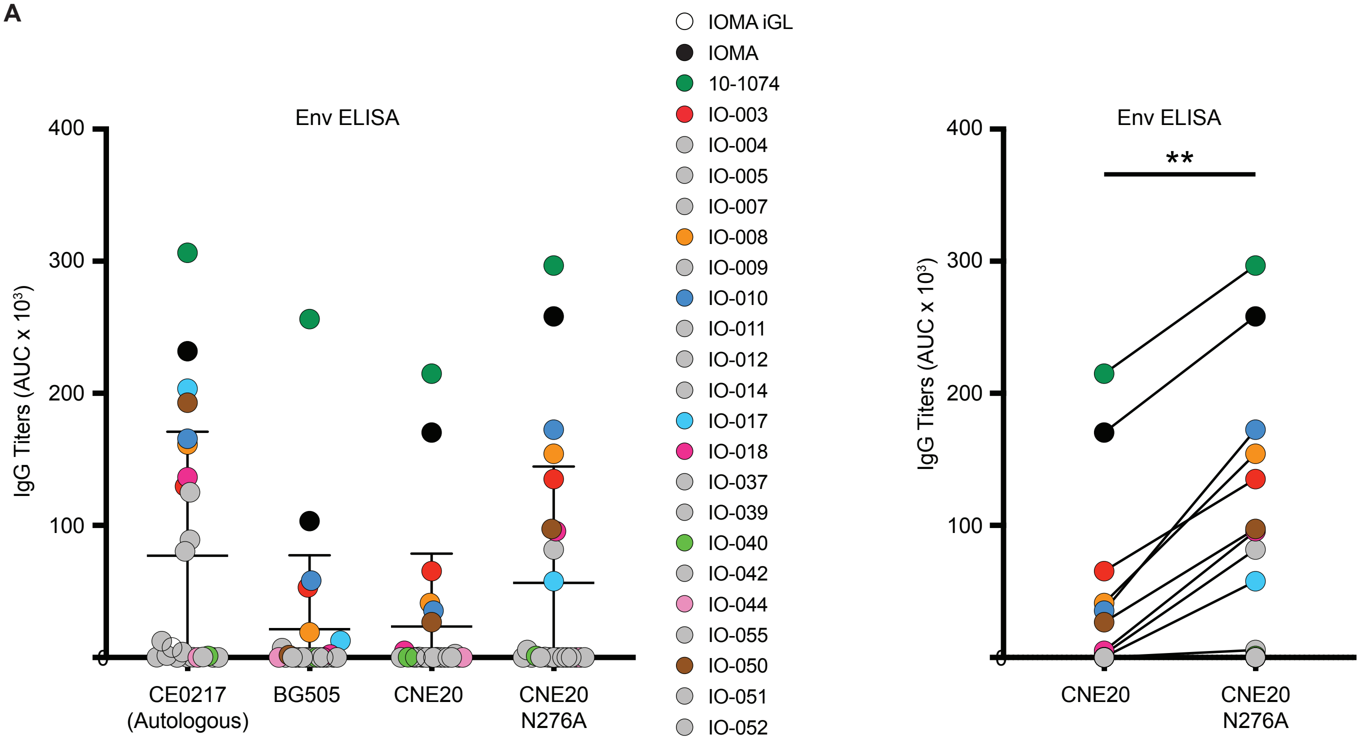


B

| Reference | | This Study | Chen et al, *Immunity* 2021 | | | |
|---|---|---|---|---|---|---|
| Immunogen | | IGT2-mi3 | eOD-GT6 60mer | | eOD-GT8 60mer | |
| Immunizations | | 4 or 5 | 6 | 9 | 4 | 9 |
| Residue | SHM | | | | | |
| Heavy Chain | | | | | | |
| K19 | T/R | 67 | N/A | N/A | N/A | N/A |
| Y33 | H/D/E/F (V/F/L/I) | 58 | 7.5 | 56 | 25 | 85 |
| V37 | (M/L/I) | N/A | 3.7 | 23 | 7.50 | 52 |
| N52 | (K/R) | N/A | 0 | 9.3 | 4.3 | 23 |
| N53 | F/Y/K/R/T (Y/K/R/T/V/L) | 73 | 31 | 56 | 20 | 65 |
| S54 | R/F/N/T (R/F/N/T/Y/W/H/G) | 82 | 41 | 65 | 46 | 88 |
| G56 | A (A) | 30 | 1.5 | 40 | 2.2 | 54 |
| T57 | V/I/P/R (V/I/P/R) | 66 | 13 | 67 | 12 | 88 |
| N58 | K/E/D | 58 | N/A | N/A | N/A | N/A |
| Q61 | (R/H/K/W) | N/A | 13 | 40 | 37 | 58 |
| V89 | (L/I/M/T) | N/A | 4.5 | 26 | 5.9 | 77 |
| Light Chain | | | | | | |
| CDR L1 | (deletion) | N/A | 2.6 | 21 | 0 | 15 |
| CDR L1 | (G/S) | N/A | 11.8 | 47 | 0 | 69 |
| Q27 | (Y/F/H) | N/A | 0 | 5.3 | 7.2 | 17 |
| S27A | R/N (F/Y) | 5.9 | 0 | 18 | 0 | 17 |
| S31 | G | 6.0 | N/A | N/A | N/A | N/A |
| Y32 | F | 10 | N/A | N/A | N/A | N/A |
| N33 | D | 42 | N/A | N/A | N/A | N/A |
| G93 | D | 33 | N/A | N/A | N/A | N/A |
| Average # Key SHMs per HC | | 4.3 | 1.6 | 3.8 | 1.6 | 6.5 |

**Figure S8. Monoclonal antibodies cloned from IOMA iGL transgenic mice bind to heterologous Envs. (A) AUC of** ELISA binding curves of selected monoclonal antibodies isolated from IOMA iGL knock-in mice to BG505, CE0217, CNE20 and CNE20 N276A SOSIPs. **(B)** Comparison of the occurrence frequency of key mutations among IOMA-like antibody sequences selected for cloning and VRC01-class antibody sequences from reference 37 at different time points throughout the respective sequential immunization regimen. Mutations essential for IOMA-class antibody binding to gp120 are listed first, while mutations essential for VRC01-class antibody binding to gp120 are listed second in brackets. Values for each residue represent the percentage of antibodies containing one of the essential mutations at that position.

## Tables S1 to S8

### Table S1: Amino acid sequences for HIV Envs and antibodies used in this study.

| Protein Name | Sequence |
|---|---|
| IGT2 gp120 | VWKEAKTTLFCASDAKAYEKECHNVWATHACVPTDPNPQEVVLENVTENFNMWKNDMVDQMQEDVISIWDQ<br>CLKPCVKLTNTSTLTQACPKVTFDPIPIHYCAPAGYAILKCNNKTFNGKGPCNNVSTVQCTHGIKPVVSTQ<br>LLLNGSLAEEEIVIRSKNLRNNAKIIIVQLNKSVEIVCTRPNNGGSGSGGDIRQAYCNISGRNWSEAVNQV<br>KKKLKEHFPHKNISFQSSSGGDLEITTHSFNCGGEFFYCNTSGLFNDTISNATIMLPCRIKQIINMWQEPG<br>KAIYAPPIKGNITCKSDITGLLLLRDGGNALRPTEIFRPSGGDMRDNWRSELYKYKVVEIKPLHHHHHH |
| IGT1 gp120 | VWKEAKTTLFCASDAKAYEKECHNVWATHACVPTDPNPQEVVLENVTENFNMWKNDMVDQMQEDVISIWDQ<br>CLKPCVKLTNTSTLTQACPKVTFDPIPIHYCAPAGYAILKCNNKTFNGKGPCNNVSTVQCTHGIKPVVSTQ<br>LLLNGSLAEEEIVIRSKNLRNNAKIIIVQLNKSVEIVCTRPNNGGSGSGGDIRQAYCNISGRNWSEAVNQV<br>KKKLKEHFPHKNISFQSSSGGDLEITTHSFNCGGEFFYCNTSGLFNDTISNATIMLPCRIKQIINMWQEPG<br>KAIYAPPIKGNITCKSDITGLLLLRDGGNSQRETEIFRPSGGDMRDNWRSELYKYKVVEIKPLHHHHHH |
| 426c.TM4 gp120 | VWKEAKTTLFCASDAKAYEKECHNVWATHACVPTDPNPQEVVLENVTENFNMWKNDMVDQMQEDVISIWDQ<br>CLKPCVKLTNTSTLTQACPKVTFDPIPIHYCAPAGYAILKCNNKTFNGKGPCNNVSTVQCTHGIKPVVSTQ<br>LLLNGSLAEEEIVIRSKNLRDNAKIIIVQLNKSVEIVCTRPNNGGSGSGGDIRQAYCNISGRNWSEAVNQV<br>KKKLKEHFPHKNISFQSSSGGDLEITTHSFNCGGEFFYCNTSGLFNDTISNATIMLPCRIKQIINMWQEVG<br>KAIYAPPIKGNITCKSDITGLLLLRDGGDTTDNTEIFRPSGGDMRDNWRSELYKYKVVEIKPLHHHHHH |
| IGT2 SOSIP | GSNLWVTVYYGVPVWKEAKTTLFCASDAKAYEKEVHNVWATHACVPTDPNPQEVVLENVTENFNMWKNDMV<br>DQMQEDVISIWDQSLKPCVKLTPLCVTLNCTNVNVTSNSTNVNSSSTDNTTLGEIKNCSFDITTEIRDKTR<br>KEYALFYRLDIVPLDNSSNPNSSNTYRLINCNTSTCTQACPKVTFDPIPIHYCAPAGYAILKCNNKTFNGK<br>GPCNNVSTVQCTHGIKPVVSTQLLLNGSLAEEEIVIRSKNLRNNAKIIIVQLNKSVEIVCTRPNNNTRRSI<br>RIGPGQTFYATDIIGDIRQAYCNISGRNWSEAVNQVKKKLKEHFPHKNISFQSSSGGDLEITTHSFNCGGE<br>FFYCNTSGLFNDTISNATIMLPCRIKQIINMWQEVGKCIYAPPIKGNITCKSDITGLLLLRDGGNALRPTE<br>IFRPSGGDMRDNWRSELYKYKVVKIEPLGVAPTRCKRRVVGRRRRRRAVGIGAVFLGFLGAAGSTMGAASM<br>TLTVQARNLLSGIVQQQSNLLRAPEAQQHLLKLTVWGIKQLQARVLAVERYLRDQQLLGIWGCSGKLICCT<br>NVPWNSSWSNRNLSEIWDNMTWLQWDKEISNYTQIIYGLLEESQNQQEKNEQDLLALD |
| IGT1 SOSIP | GSNLWVTVYYGVPVWKEAKTTLFCASDAKAYEKEVHNVWATHACVPTDPNPQEVVLENVTENFNMWKNDMV<br>DQMQEDVISIWDQSLKPCVKLTPLCVTLNCTNVNVTSNSTNVNSSSTDNTTLGEIKNCSFDITTEIRDKTR<br>KEYALFYRLDIVPLDNSSNPNSSNTYRLINCNTSTCTQACPKVTFDPIPIHYCAPAGYAILKCNNKTFNGK<br>GPCNNVSTVQCTHGIKPVVSTQLLLNGSLAEEEIVIRSKNLRNNAKIIIVQLNKSVEIVCTRPNNNTRRSI<br>RIGPGQTFYATDIIGDIRQAYCNISGRNWSEAVNQVKKKLKEHFPHKNISFQSSSGGDLEITTHSFNCGGE<br>FFYCNTSGLFNDTISNATIMLPCRIKQIINMWQEVGKCIYAPPIKGNITCKSDITGLLLLRDGGNSQRETE<br>IFRPSGGDMRDNWRSELYKYKVVKIEPLGVAPTRCKRRVVGRRRRRRAVGIGAVFLGFLGAAGSTMGAASM<br>TLTVQARNLLSGIVQQQSNLLRAPEAQQHLLKLTVWGIKQLQARVLAVERYLRDQQLLGIWGCSGKLICCT<br>NVPWNSSWSNRNLSEIWDNMTWLQWDKEISNYTQIIYGLLEESQNQQEKNEQDLLALD |
| 426c SOSIP | AENLWVTVYYGVPVWKEAKTTLFCASDAKAYEKEVHNVWATHACVPTDPNPQEVVLENVTENFNMWKNDMV<br>DQMQEDVISIWDQSLKPCVKLTPLCVTLNCTNVNVTSNSTNVNSSSTDNTTLGEIKNCSFDITTEIRDKTR<br>KEYALFYRLDIVPLDNSSNPNSSNTYRLINCNTSTCTQACPKVTFDPIPIHYCAPAGYAILKCNNKTFNGK<br>GPCNNVSTVQCTHGIKPVVSTQLLLNGSLAEEEIVIRSKNLSDNAKIIIVQLNKSVEIVCTRPNNNTRRSI<br>RIGPGQTFYATDIIGDIRQAYCNISGRNWSEAVNQVKKKLKEHFPHKNISFQSSSGGDLEITTHSFNCGGE<br>FFYCNTSGLFNDTISNATIMLPCRIKQIINMWQEVGKCIYAPPIKGNITCKSDITGLLLLRDGGNTTNNTE<br>IFRPGGGDMRDNWRSELYKYKVVKIEPLGVAPTRCKRRVVGRRRRRRAVGIGAVFLGFLGAAGSTMGAASM<br>TLTVQARNLLSGIVQQQSNLLRAPEAQQHLLKLTVWGIKQLQARVLAVERYLRDQQLLGIWGCSGKLICCT<br>NVPWNSSWSNRNLSEIWDNMTWLQWDKEISNYTQIIYGLLEESQNQQEKNEQDLLALD |
| 426c D279N SOSIP | AENLWVTVYYGVPVWKEAKTTLFCASDAKAYEKEVHNVWATHACVPTDPNPQEVVLENVTENFNMWKNDMV<br>DQMQEDVISIWDQSLKPCVKLTPLCVTLNCTNVNVTSNSTNVNSSSTDNTTLGEIKNCSFDITTEIRDKTR<br>KEYALFYRLDIVPLDNSSNPNSSNTYRLINCNTSTCTQACPKVTFDPIPIHYCAPAGYAILKCNNKTFNGK<br>GPCNNVSTVQCTHGIKPVVSTQLLLNGSLAEEEIVIRSKNLSNNAKIIIVQLNKSVEIVCTRPNNNTRRSI<br>RIGPGQTFYATDIIGDIRQAYCNISGRNWSEAVNQVKKKLKEHFPHKNISFQSSSGGDLEITTHSFNCGGE<br>FFYCNTSGLFNDTISNATIMLPCRIKQIINMWQEVGKCIYAPPIKGNITCKSDITGLLLLRDGGNTTNNTE<br>IFRPGGGDMRDNWRSELYKYKVVKIEPLGVAPTRCKRRVVGRRRRRRAVGIGAVFLGFLGAAGSTMGAASM<br>TLTVQARNLLSGIVQQQSNLLRAPEAQQHLLKLTVWGIKQLQARVLAVERYLRDQQLLGIWGCSGKLICCT<br>NVPWNSSWSNRNLSEIWDNMTWLQWDKEISNYTQIIYGLLEESQNQQEKNEQDLLALD |

| | |
|---|---|
| 426c degly2 SOSIP | AENLWVTVYYGVPVWKEAKTTLFCASDAKAYEKEVHNVWATHACVPTDPNPQEVVLENVTENFNMWKNDMV<br>DQMQEDVISIWDQSLKPCVKLTPLCVTLNCTNVNVTSNSTNVNSSSTDNTTLGEIKNCSFDITTEIRDKTR<br>KEYALFYRLDIVPLDNSSNPNSSNTYRLINCNTSTCTQACPKVTFDPIPIHYCAPAGYAILKCNNKTFNGK<br>GPCNNVSTVQCTHGIKPVVSTQLLLNGSLAEEEIVIRSKNLTDNAKIIIVQLNKSVEIVCTRPNNNTRRSI<br>RIGPGQTFYATDIIGDIRQAYCNISGRNWSEAVNQVKKKLKEHFPHKNISFQSSSGGDLEITTHSFNCGGE<br>FFYCNTSGLFNDTISNATIMLPCRIKQIINMWQEVGKCIYAPPIKGNITCKSDITGLLLLRDGGNTANNAE<br>IFRPGGGDMRDNWRSELYKYKVVKIEPLGVAPTRCKRRVVGRRRRRRAVGIGAVFLGFLGAAGSTMGAASM<br>TLTVQARNLLSGIVQQQSNLLRAPEAQQHLLKLTVWGIKQLQARVLAVERYLRDQQLLGIWGCSGKLICCT<br>NVPWNSSWSNRNLSEIWDNMTWLQWDKEISNYTQIIYGLLEESQNQQEKNEQDLLALD |
| 426c degly2 D279N SOSIP | AENLWVTVYYGVPVWKEAKTTLFCASDAKAYEKEVHNVWATHACVPTDPNPQEVVLENVTENFNMWKNDMV<br>DQMQEDVISIWDQSLKPCVKLTPLCVTLNCTNVNVTSNSTNVNSSSTDNTTLGEIKNCSFDITTEIRDKTR<br>KEYALFYRLDIVPLDNSSNPNSSNTYRLINCNTSTCTQACPKVTFDPIPIHYCAPAGYAILKCNNKTFNGK<br>GPCNNVSTVQCTHGIKPVVSTQLLLNGSLAEEEIVIRSKNLTNNAKIIIVQLNKSVEIVCTRPNNNTRRSI<br>RIGPGQTFYATDIIGDIRQAYCNISGRNWSEAVNQVKKKLKEHFPHKNISFQSSSGGDLEITTHSFNCGGE<br>FFYCNTSGLFNDTISNATIMLPCRIKQIINMWQEVGKCIYAPPIKGNITCKSDITGLLLLRDGGNTANNAE<br>IFRPGGGDMRDNWRSELYKYKVVKIEPLGVAPTRCKRRVVGRRRRRRAVGIGAVFLGFLGAAGSTMGAASM<br>TLTVQARNLLSGIVQQQSNLLRAPEAQQHLLKLTVWGIKQLQARVLAVERYLRDQQLLGIWGCSGKLICCT<br>NVPWNSSWSNRNLSEIWDNMTWLQWDKEISNYTQIIYGLLEESQNQQEKNEQDLLALD |
| 426c degly3 SOSIP | AENLWVTVYYGVPVWKEAKTTLFCASDAKAYEKEVHNVWATHACVPTDPNPQEVVLENVTENFNMWKNDMV<br>DQMQEDVISIWDQSLKPCVKLTPLCVTLNCTNVNVTSNSTNVNSSSTDNTTLGEIKNCSFDITTEIRDKTR<br>KEYALFYRLDIVPLDNSSNPNSSNTYRLINCNTSTCTQACPKVTFDPIPIHYCAPAGYAILKCNNKTFNGK<br>GPCNNVSTVQCTHGIKPVVSTQLLLNGSLAEEEIVIRSKALTDNAKIIIVQLNKSVEIVCTRPNNNTRRSI<br>RIGPGQTFYATDIIGDIRQAYCNISGRNWSEAVNQVKKKLKEHFPHKNISFQSSSGGDLEITTHSFNCGGE<br>FFYCNTSGLFNDTISNATIMLPCRIKQIINMWQEVGKCIYAPPIKGNITCKSDITGLLLLRDGGNTANNAE<br>IFRPGGGDMRDNWRSELYKYKVVKIEPLGVAPTRCKRRVVGRRRRRRAVGIGAVFLGFLGAAGSTMGAASM<br>TLTVQARNLLSGIVQQQSNLLRAPEAQQHLLKLTVWGIKQLQARVLAVERYLRDQQLLGIWGCSGKLICCT<br>NVPWNSSWSNRNLSEIWDNMTWLQWDKEISNYTQIIYGLLEESQNQQEKNEQDLLALD |
| BG505.v4.1-GT1 SOSIP | AENLWVTVYYGVPVWKDAETTLFCASDAKAYETKKHNVWATHACVPTDPNPQEIHLENVTEEFNMWKNNMV<br>EQMHTDIISLWDQSLKPCVKLTPLCVTLQCTNVTNAITDDMRGELKNCSFNMTTELRDKRQKVHALFYKLD<br>IVPINENQNTSYRLINCNTAAITQACPKVSFEPIPIHYCAPAGFAILKCKDKKFNGTGPCPSVSTVQCTHG<br>IKPVVSTQLLLNGSLAEEEVMIRSEDIRNNAKNILVQFNTPVQINCTRPNNNTRKSIRIGPGQWFYATGDI<br>IGDIRQAHCNVSKATWNETLGKVVKQLRKHFGNNTIIRFANSSGGDLEVTTHSFNCGGEFFYCDTSGLFNS<br>TWISNTSVQGSNSTGSNDSITLPCRIKQIINMWQRIGQAMYAPPIQGVIRCVSNITGLILTRDGGSTDSTT<br>ETFRPSGGDMRDNWRSELYKYKVVKIEPLGVAPTRCKRRVVGRRRRRRAVGIGAVFLGFLGAAGSTMGAAS<br>MTLTVQARNLLSGIVQQQSNLLRAPEAQQHLLKLTVWGIKQLQARVLAVERYLRDQQLLGIWGCSGKLICC<br>TNVPWNSSWSNRNLSEIWDNMTWLQWDKEISNYTQIIYGLLEESQNQQEKNEQDLLALD |
| eOD-GT8 | DTITLPCRPAPPPHCSSNITGLILTRQGGYSNANTVIFRPSGGDWRDIARCQIAGTVVSTQLFLNGSLAEE<br>EVVIRSEDWRDNAKSICVQLATSVEIACTGAGHCAISRAKWANTLKQIASKLREQYGAKTIIFKPSSGGDP<br>EFVNHSFNCGGEFFYCASTQLFASTWFASTGTGTK |
| IGT2 SOSIP SpyTag | GSNLWVTVYYGVPVWKEAKTTLFCASDAKAYEKEVHNVWATHACVPTDPNPQEVVLENVTENFNMWKNDMV<br>DQMQEDVISIWDQSLKPCVKLTPLCVTLNCTNVNVTSNSTNVNSSSTDNTTLGEIKNCSFDITTEIRDKTR<br>KEYALFYRLDIVPLDNSSNPNSSNTYRLINCNTSTCTQACPKVTFDPIPIHYCAPAGYAILKCNNKTFNGK<br>GPCNNVSTVQCTHGIKPVVSTQLLLNGSLAEEEIVIRSKNLRNNAKIIIVQLNKSVEIVCTRPNNNTRRSI<br>RIGPGQTFYATDIIGDIRQAYCNISGRNWSEAVNQVKKKLKEHFPHKNISFQSSSGGDLEITTHSFNCGGE<br>FFYCNTSGLFNDTISNATIMLPCRIKQIINMWQEVGKCIYAPPIKGNITCKSDITGLLLLRDGGNALRPTE<br>IFRPSGGDMRDNWRSELYKYKVVKIEPLGVAPTRCKRRVVGRRRRRRAVGIGAVFLGFLGAAGSTMGAASM<br>TLTVQARNLLSGIVQQQSNLLRAPEAQQHLLKLTVWGIKQLQARVLAVERYLRDQQLLGIWGCSGKLICCT<br>NVPWNSSWSNRNLSEIWDNMTWLQWDKEISNYTQIIYGLLEESQNQQEKNEQDLLALDGGGGSGGGSGGGS<br>GSGAHIVMVDAYKPTK |
| IGT1 SOSIP SpyTag | GSNLWVTVYYGVPVWKEAKTTLFCASDAKAYEKEVHNVWATHACVPTDPNPQEVVLENVTENFNMWKNDMV<br>DQMQEDVISIWDQSLKPCVKLTPLCVTLNCTNVNVTSNSTNVNSSSTDNTTLGEIKNCSFDITTEIRDKTR<br>KEYALFYRLDIVPLDNSSNPNSSNTYRLINCNTSTCTQACPKVTFDPIPIHYCAPAGYAILKCNNKTFNGK<br>GPCNNVSTVQCTHGIKPVVSTQLLLNGSLAEEEIVIRSKNLRNNAKIIIVQLNKSVEIVCTRPNNNTRRSI<br>RIGPGQTFYATDIIGDIRQAYCNISGRNWSEAVNQVKKKLKEHFPHKNISFQSSSGGDLEITTHSFNCGGE<br>FFYCNTSGLFNDTISNATIMLPCRIKQIINMWQEVGKCIYAPPIKGNITCKSDITGLLLLRDGGNSQRETE<br>IFRPSGGDMRDNWRSELYKYKVVKIEPLGVAPTRCKRRVVGRRRRRRAVGIGAVFLGFLGAAGSTMGAASM<br>TLTVQARNLLSGIVQQQSNLLRAPEAQQHLLKLTVWGIKQLQARVLAVERYLRDQQLLGIWGCSGKLICCT<br>NVPWNSSWSNRNLSEIWDNMTWLQWDKEISNYTQIIYGLLEESQNQQEKNEQDLLALDGGGGSGGGSGGGS<br>GSGAHIVMVDAYKPTK |

| | |
|---|---|
| 426c degly2<br>D279N SOSIP<br>SpyTag | AENLWVTVYYGVPVWKEAKTTLFCASDAKAYEKEVHNVWATHACVPTDPNPQEVVLENVTENFNMWKNDMV<br>DQMQEDVISIWDQSLKPCVKLTPLCVTLNCTNVNVTSNSTNVNSSSTDNTTLGEIKNCSFDITTEIRDKTR<br>KEYALFYRLDIVPLDNSSNPNSSNTYRLINCNTSTCTQACPKVTFDPIPIHYCAPAGYAILKCNNKTFNGK<br>GPCNNVSTVQCTHGIKPVVSTQLLLNGSLAEEEIVIRSKNLTNNAKIIIVQLNKSVEIVCTRPNNNTRRSI<br>RIGPGQTFYATDIIGDIRQAYCNISGRNWSEAVNQVKKKLKEHFPHKNISFQSSSGGDLEITTHSFNCGGE<br>FFYCNTSGLFNDTISNATIMLPCRIKQIINMWQEVGKCIYAPPIKGNITCKSDITGLLLLRDGGNTANNAE<br>IFRPGGGDMRDNWRSELYKYKVVKIEPLGVAPTRCKRRVVGRRRRRRAVGIGAVFLGFLGAAGSTMGAASM<br>TLTVQARNLLSGIVQQQSNLLRAPEAQQHLLKLTVWGIKQLQARVLAVERYLRDQQLLGIWGCSGKLICCT<br>NVPWNSSWSNRNLSEIWDNMTWLQWDKEISNYTQIIYGLLEESQNQQEKNEQDLLALDGGGGSGGGSGGGS<br>GSGRGVPHIVMVDAYKRYK |
| 398F1 SOSIP<br>SpyTag | AENLWVTVYYGVPVWKDAETTLFCASDAKAYHTEVHNVWATHACVPTDPNPQEINLENVTEEFNMWKNKMV<br>EQMHTDIISLWDQSLKPCVQLTPLCVTLDCQYNVTNINSTSDMAREINNCSYNITTELRDREQKVYSLFYR<br>SDIVQMNSDNSSKYRLINCNTSACKQACPKVTFEPIPIHYCAPAGFAILKCKDKEFNGTGPCKNVSTVQCT<br>HGIKPVVSTQLLLNGSLAEEKVMIRSENITDNAKNIIVQFKEPVKINCTRPNNNTRKSVRIGPGQTFYATG<br>EIIGDIRQAHCNVSKAHWENTLQEVANQLKLMIHSNKTIIFANSSGGDLEITTHSFNCGGEFFYCYTSGLF<br>NYTFNDTSTNSTESKSNDTITLQCRIKQIINMWQRAGQCVYAPPIPGIIRCESNITGLILTRDGGNNNSNT<br>NETFRPGGGDMRDNWRSELYRYKVVKIEPLGVAPTRCKRRVVGRRRRRRAVGIGAVSLGFLGAAGSTMGAA<br>SMTLTVQARNLLSGIVQQQSNLLRAPEPQQHLLKDTHWGIKQLQARVLAVEHYLRDQQLLGIWGCSGKLIC<br>CTNVPWNSSWSNRNLSEIWDNMTWLQWDKEISNYTQIIYGLLEESQNQQEKNEQDLLALDGGGGSGGGSGG<br>GSGSGAHIVMVDAYKPTK |
| BJOX2000 SOSIP<br>SpyTag | AENLWVTVYYGVPVWKEATTTLFCASDAKAYDTEVHNVWATHACVPTDPDPQEMFLENVTENFNMWKNNMV<br>DQMHEDVISLWDQSLKPCVKLTPLCVTLECKNVNSSSSDTKNGTDPEMKNCSFNATTELRDRKQKVYALFY<br>KLDIVPLNEKNSSEYRLINCNTSTCTQACPKVTFDPIPIHYCTPAGYAILKCNDEKFNGTGPCSNVSTVQC<br>THGIKPVVSTQLLLNGSLAEKGIVIRSENLTNNVKTIIVHLNQSVEILCIRPNNNTRKSIRIGPGQTFYAT<br>GEIIGDIRQAHCNISGKVWNETLQRVGEKLAEYFPNKTIKFNSSSGGDLEITTHSFNCGGEFFYCNTSKLF<br>NGTFNGTYMPNVTEGNSTISIPCRIKQIINMWQKVGRCMYAPPIEGNITCKSKITGLLLERDGGPENDTEI<br>FRPGGGDMRNNWRSELYKYKVVEIKPLGVAPTRCKRRVVGRRRRRRAVGIGAVSLGFLGAAGSTMGAASMT<br>LTVQARNLLSGIVQQQSNLLRAPEPQQHLLKDTHWGIKQLQARVLAVEHYLRDQQLLGIWGCSGKLICCTN<br>VPWNSSWSNRNLSEIWDNMTWLQWDKEISNYTQIIYGLLEESQNQQEKNEQDLLALDGGGGSGGGSGGGSG<br>SGAHIVMVDAYKPTK |
| CE1176 SOSIP<br>SpyTag | AENLWVTVYYGVPVWKEAKTTLFCASDAKAYEKEVHNVWATHACVPTDPNPQEMVLENVTENFNMWKNDMV<br>DQMHEDVISLWDQSLKPCVKLTPLCVTLTCTNTTVSNGSSNSNANFEEMKNCSFNATTEIKDKKKNEYALF<br>YKLDIVPLNNSSGKYRLINCNTSACAQACPKVTFEPIPIHYCAPAGYAILKCNNKTFNGTGPCNNVSTVQC<br>THGIKPVVSTQLLLNGSLAEKEIIIRSENLTNNAKTIIIHFNESVGIVCTRPSNNTRKSIRIGPGQTFYAT<br>GDIIGDIRQAHCNVSKQNWNRTLQQVGRKLAEHFPNRNITFNHSSGGDLEITTHSFNCRGEFFYCNTSGLF<br>NGTYHPNGTYNETAVNSSDTITLQCRIKQIINMWQEVGRCMYAPPIAGNITCNSTITGLLLTRDGGINQTG<br>EEIFRPGGGDMRDNWRNELYKYKVVEIKPLGVAPTRCKRRVVGRRRRRRAVGIGAVSLGFLGAAGSTMGAA<br>SMTLTVQARNLLSGIVQQQSNLLRAPEPQQHLLKDTHWGIKQLQARVLAVEHYLRDQQLLGIWGCSGKLIC<br>CTNVPWNSSWSNRNLSEIWDNMTWLQWDKEISNYTQIIYGLLEESQNQQEKNEQDLLALDGGGGSGGGSGG<br>GSGSGAHIVMVDAYKPTK |
| CE0217 SOSIP<br>SpyTag | AENLWVTVYYGVPVWREAKTTLFCASDAKAYEREVHNVWATHACVPTDPNPQERVLENVTENFNMWKNNMV<br>DQMHEDIISLWDESLKPCIKLTPLCVTLNCGNAIVNESTIEGMKNCSFNVTTELKDKKKKEYALFYKLDVV<br>PLNGENNNSNSKNFSEYRLINCNTSTCTQACPKVSFDPIPIHYCAPAGFAILKCNNETFNGTGPCNNVSTV<br>QCTHGIKPVVSTQLLLNGSLAEKEIIIRSENLTNNAKIIIVHLNNPVKIICTRPGNNTRKSMRIGPGQTFY<br>ATGDIIGDIRRAYCNISEKTWYDTLKNVSDKFQEHFPNASIEFKPSAGGDLEITTHSFNCRGEFFYCDTSE<br>LFNGTYNNSTYNSSNNITLQCKIKQIINMWQGVGRCMYAPPIAGNITCESNITGLLLTRDGGNNKSTPETF<br>RPGGGDMRDNWRSELYKYKVVEIKPLGVAPTRCKRRVVGRRRRRRAVGIGAVSLGFLGAAGSTMGAASMTL<br>TVQARNLLSGIVQQQSNLLRAPEPQQHLLKDTHWGIKQLQARVLAVEHYLRDQQLLGIWGCSGKLICCTNV<br>PWNSSWSNRNLSEIWDNMTWLQWDKEISNYTQIIYGLLEESQNQQEKNEQDLLALDGGGGSGGGSGGGSGS<br>GAHIVMVDAYKPTK |
| CNE55 SOSIP<br>SpyTag | AENLWVTVYYGVPVWRDADTTLFCASDAKAHETEVHNVWATHACVPTDPNPQEIHLVNVTENFNMWKNKMV<br>EQMQEDVISLWDESLKPCVKLTPLCVTLNCTTANTNETKNNTTDDNIKDEMKNCTFNMTTEIRDKKQRVSA<br>LFYKLDIVPIDDSKNNSEYRLINCNTSVCKQACPKVSFDPIPIHYCTPAGYVILKCNDKNFNGTGPCKNVS<br>SVQCTHGIKPVVSTQLLLNGSLAEEEIIIRSENLTDNAKNIIVHLNKSVEINCTRPSNNTRTSVRIGPGQV<br>FYRTGDITGDIRKAYCNISGTEWNKTLTQVAEKLKEHFNKTIVYQPPSGGDLEITMHHFNCRGEFFYCNTT<br>QLFNNSVGNSTIKLPCRIKQIINMWQGVGQCMYAPPISGAINCLSNITGILLTRDGGGNNRSNETFRPGGG<br>NIKDNWRSELYKYKVVEIEPLGVAPTRCKRRVVGRRRRRRAVGIGAVSLGFLGAAGSTMGAASMTLTVQAR<br>NLLSGIVQQQSNLLRAPEPQQHLLKDTHWGIKQLQARVLAVEHYLRDQQLLGIWGCSGKLICCTNVPWNSS<br>WSNRNLSEIWDNMTWLQWDKEISNYTQIIYGLLEESQNQQEKNEQDLLALDGGGGSGGGSGGGSGSGAHIV<br>MVDAYKPTK |

| | |
|---|---|
| Tro11 SOSIP SpyTag | AENLWVTVYYGVPVWKDASTTLFCASDAKAYDTEVHNVWATHACVPTDPNPQEVVLGNVTENFNMWKNNMV<br>DQMHEDIISLWDQSLKPCVKLTPLCVTLNCTDNITNTNTNSSKNSSTHSYNNSLEGEMKNCSFNITAGIRD<br>KVKKEYALFYKLDVVPIEEDKDTNKTTYRLRSCNTSVCTQACPKVTFEPIPIHYCAPAGFAILKCNDKKFN<br>GTGPCTNVSTVQCTHGIRPVVSTQLLLNGSLAEEEVVIRSENFTNNAKTIIVQLNESIAINCTRPNNNTRR<br>SIHIGPGRAFYATGDIIGDIRQAHCNISRTEWNSTLRQIVTKLREQLGDPNKTIIFNQSSGGDTEITMHSF<br>NCGGEFFYCNTTKLFNSTWNGNNTTESDSTGENITLPCRIKQIINLWQEVGKCMYAPPIKGQISCSSNITG<br>LLLTRDGGNNNSSGPETFRPGGGNMKDNWRSELYKYKVIKIEPLGVAPTRCKRRVVGRRRRRRAVGIGAVS<br>LGFLGAAGSTMGAASMTLTVQARNLLSGIVQQQSNLLRAPEPQQHLLKDTHWGIKQLQARVLAVEHYLRDQ<br>QLLGIWGCSGKLICCTNVPWNSSWSNRNLSEIWDNMTWLQWDKEISNYTQIIYGLLEESQNQQEKNEQDLL<br>ALDGGGGSGGGSGGGSGSGAHIVMVDAYKPTK |
| X1632 SOSIP SpyTag | AENLWVTVYYGVPVWEDADTTLFCASDAKAYSTESHNVWATHACVPTDPNPQEIYLENVTEDFNMWENNMV<br>EQMQEDIISLWDESLKPCVKLTPLCVTLTCTNVTNVTDSVGTNSRLKGYKEELKNCSFNTTTEIRDKKKQE<br>YALFYKLDIVPINDNSNNSNGYRLINCNVSTCKQACPKVSFDPIPIHYCAPAGFAILKCRDKEFNGTGTCR<br>NVSTVQCTHGIKPVVSTQLLLNGSLAEGDIVIRSENITDNAKTIIVHLNKTVSITCTRPNNNTRKSIRIGP<br>GQALYATGAIIGDTRQAHCNISGSEWYEMIQNVKNKLNETFKKNITFNPSSGGDLEITTHSFNCRGEFFYC<br>NTSELFNSSHLFNGSTLSTNGTITLPCRIKQIVRMWQRVGQCMYAPPIAGNITCRSNITGLLLTRDGGTNK<br>DTNEAETFRPGGGDMRDNWRSELYKYKVVKIKPLGVAPTRCKRRVVGRRRRRRAVGIGAVSLGFLGAAGST<br>MGAASMTLTVQARNLLSGIVQQQSNLLRAPEPQQHLLKDTHWGIKQLQARVLAVEHYLRDQQLLGIWGCSG<br>KLICCTNVPWNSSWSNRNLSEIWDNMTWLQWDKEISNYTQIIYGLLEESQNQQEKNEQDLLALDGGGGSGG<br>GSGGGSGSGAHIVMVDAYKPTK |
| X2278 SOSIP SpyTag | AENLWVTVYYGVPVWKEATTTLFCASEAKAYDTEVHNIWATHACVPTDPNPQEMELKNVTENFNMWKNNMV<br>EQMHQDIISLWDQSLKPCVKLTPLCVTLDCTNINSTNSTNNTSSNSKMEETIGVIKNCSFNVTTNIRDKVK<br>KENALFYSLDLVSIGNSNTSYRLISCNTSICTQACPKVSFDPIPIHYCAPAGFAILKCRDKKFNGTGPCRN<br>VSSVQCTHGIRPVVSTQLLLNGSLAEEEIVIRSANLTDNAKTIIIQLNETIQINCTRPNNNTRRSIPIGPG<br>RTFYATGDIIGDIRKAYCNISATKWNNTLRQIAEKLREKFNKTIIFNQSSGGDPEVVRHTFNCGGEFFYCN<br>SSQLFNSTWYSNGTSNGGLNNSANITLPCRIKQIINLWQEVGKCMYAPPIKGVINCLSNITGIILTRDGGE<br>NNGTTETFRPGGGDMRDNWRSELYKYKVVKIEPLGVAPTRCKRRVVGRRRRRRAVGIGAVSLGFLGAAGST<br>MGAASMTLTVQARNLLSGIVQQQSNLLRAPEPQQHLLKDTHWGIKQLQARVLAVEHYLRDQQLLGIWGCSG<br>KLICCTNVPWNSSWSNRNLSEIWDNMTWLQWDKEISNYTQIIYGLLEESQNQQEKNEQDLLALDGGGGSGG<br>GSGGGSGSGAHIVMVDAYKPTK |
| BG505 SOSIP | NLWVTVYYGVPVWKDAETTLFCASDAKAYETEKHNVWATHACVPTDPNPQEIHLENVTEEFNMWKNNMVEQ<br>MHTDIISLWDQSLKPCVKLTPLCVTLQCTNVTNNITDDMRGELKNCSFNMTTELRDKKQKVYSLFYRLDVV<br>QINENQGNRSNNSNKEYRLINCNTSAITQACPKVSFEPIPIHYCAPAGFAILKCKDKKFNGTGPCPSVSTV<br>QCTHGIKPVVSTQLLLNGSLAEEEVMIRSENITNNAKNILVQFNTPVQINCTRPNNNTRKSIRIGPGQAFY<br>ATGDIIGDIRQAHCNVSKATWNETLGKVVKQLRKHFGNNTIIRFANSSGGDLEVTTHSFNCGGEFFYCNTS<br>GLFNSTWISNTSVQGSNSTGSNDSITLPCRIKQIINMWQRIGQAMYAPPIQGVIRCVSNITGLILTRDGGS<br>TNSTTETFRPGGGDMRDNWRSELYKYKVVKIEPLGVAPTRCKRRVVGRRRRRRAVGIGAVSLGFLGAAGST<br>MGAASMTLTVQARNLLSGIVQQQSNLLRAPEPQQHLLKDTHWGIKQLQARVLAVEHYLRDQQLLGIWGCSG<br>KLICCTNVPWNSSWSNRNLSEIWDNMTWLQWDKEISNYTQIIYGLLEESQNQQEKNEQDLLALD |
| AMC011 SOSIP | AEQLWVTVYYGVPVWKEATTTLFCASDARAYDTEVRNVWATHCCVPTDPNPQEVVLENVTENFNMWKNNMV<br>EQMHEDIISLWDQSLKPCVKLTPLCVTLNCTDLRNATNTNATNTTSSSRGTMEGGEIKNCSFNITTSMRDK<br>VQKEYALFYKLDVVPIKNDNTSYRLISCNTSVITQACPKVSFEPIPIHYCAPAGFAILKCNNKTFNGTGPC<br>TNVSTVQCTHGIRPVVSTQLLLNGSLAEEEVVIRSANFTDNAKIIIVQLNKSVEINCTRPNNNTRKSIHIG<br>PGRWFYTTGEIIGDIRQAHCNISGTKWNDTLKQIVVKLKEQFGNKTIVFNHSSGGDPEIVMHSFNCGGEFF<br>YCNSTQLFNSTWNDTTGSNYTGTIVLPCRIKQIVNMWQEVGKAMYAPPIKGQIRCSSNITGLILIRDGGKN<br>RSENTEIFRPGGGDMRDNWRSELYKYKVVKIEPLGIAPTKCKRRVVQRRRRRRAVGIGAVFLGFLGAAGST<br>MGAASMTLTVQARQLLSGIVQQQNNLLRAPECQQHLLKLTVWGIKQLQARVLAVERYLKDQQLLGIWGCSG<br>KLICCTAVPWNTSWSNKSYNQIWNNMTWMEWEREIDNYTSLIYTLIEDSQNQQEKNEQELLELD |
| B41 SOSIP | AAKKWVTVYYGVPVWKEATTTLFCASDAKAYDTEVHNVWATHACVPTDPNPQEIVLGNVTENFNMWKNNMV<br>EQMHEDIISLWDQSLKPCVKLTPLCVTLNCNNVNTNNTNNSTNATISDWEKMETGEMKNCSFNVTTSIRDK<br>IKKEYALFYKLDVVPLENKNNINNTNITNYRLINCNTSVITQACPKVSFEPIPIHYCAPAGFAILKCNSKT<br>FNGSGPCTNVSTVQCTHGIRPVVSTQLLLNGSLAEEEIVIRSENITDNAKTIIVQLNEAVEINCTRPNNNT<br>RKSIHIGPGRWFYATGDIIGNIRQAHCNISKARWNETLGQIVAKLEEQFPNKTIIFNHSSGGDPEIVTHSF<br>NCGGEFFYCNTTPLFNSTWNNTRTDDYPTGGEQNITLQCRIKQIINMWQGVGKAMYAPPIRGQIRCSSNIT<br>GLLLTRDGGRDQNGTETFRPGGGNMRDNWRSELYKYKVVKIEPLGIAPTACKRRVVQRRRRRRAVGLGAFI<br>LGFLGAAGSTMGAASMALTVQARLLLSGIVQQQNNLLRAPEAQQHMLQLTVWGIKQLQARVLAVERYLRDQ<br>QLLGIWGCSGKIICCTNVPWNDSWSNKTINEIWDNMTWMQWEKEIDNYTQHIYTLLEVSQIQQEKNEQELL<br>ELD |

| | |
|---|---|
| CH119 SOSIP | AENLWVTVYYGVPVWKEATTTLFCASDAKAYDTEVHNVWATHACVPTDPSPQELVLENVTENFNMWKNEMV<br>NQMHEDVISLWDQSLKPCVKLTPLCVTLECSKVSNNETDKYNGTEEMKNCSFNATTVVRDRQQKVYALFYR<br>LDIVPLTEKNSSENSSKYYRLINCNTSACTQACPKVSFEPIPIHYCTPAGYAILKCNDKTFNGTGPCHNVS<br>TVQCTHGIKPVVSTQLLLNGSLAEGEIIIRSENLTNNVKTILVHLNQSVEIVCTRPNNNTRKSIRIGPGQT<br>FYATGDIIGDIRQAHCNISKWHETLKRVSEKLAEHFPNKTINFTSSSGGDLEITTHSFTCRGEFFYCNTSG<br>LFNSTYMPNGTYLHGDTNSNSSITIPCRIKQIINMWQEVGRCMYAPPIEGNITCKSNITGLLLVRDGGTES<br>NNTETNNTEIFRPGGGDMRDNWRSELYKYKVVEIKPLGVAPTRCKRRVVGRRRRRRAVGIGAVSLGFLGAA<br>GSTMGAASMTLTVQARNLLSGIVQQQSNLLRAPEPQQHLLKDTHWGIKQLQARVLAVEHYLRDQQLLGIWG<br>CSGKLICCTNVPWNSSWSNRNLSEIWDNMTWLQWDKEISNYTQIIYGLLEESQNQQEKNEQDLLALD |
| CE0217 SOSIP | AENLWVTVYYGVPVWREAKTTLFCASDAKAYEREVHNVWATHACVPTDPNPQERVLENVTENFNMWKNNMV<br>DQMHEDIISLWDESLKPCIKLTPLCVTLNCGNAIVNESTIEGMKNCSFNVTTELKDKKKKEYALFYKLDVV<br>PLNGENNNSNSKNFSEYRLINCNTSTCTQACPKVSFDPIPIHYCAPAGFAILKCNNETFNGTGPCNNVSTV<br>QCTHGIKPVVSTQLLLNGSLAEKEIIIRSENLTNNAKIIIVHLNNPVKIICTRPGNNTRKSMRIGPGQTFY<br>ATGDIIGDIRRAYCNISEKTWYDTLKNVSDKFQEHFPNASIEFKPSAGGDLEITTHSFNCRGEFFYCDTSE<br>LFNGTYNNSTYNSSNNITLQCKIKQIINMWQGVGRCMYAPPIAGNITCESNITGLLLTRDGGNNKSTPETF<br>RPGGGDMRDNWRSELYKYKVVEIKPLGVAPTRCKRRVVGRRRRRRAVGIGAVSLGFLGAAGSTMGAASMTL<br>TVQARNLLSGIVQQQSNLLRAPEPQQHLLKDTHWGIKQLQARVLAVEHYLRDQQLLGIWGCSGKLICCTNV<br>PWNSSWSNRNLSEIWDNMTWLQWDKEISNYTQIIYGLLEESQNQQEKNEQDLLALD |
| CNE8 SOSIP | AENLWVTVYYGVPVWRDADTTLFCASDAKAYDTEVHNVWATHACVPTDPNPQEIHLENVTENFNMWKNKMA<br>EQMQEDVISLWDESLKPCVQLTPLCVTLNCTNANLNATVNASTTIGNITDEVRNCSFNTTTELRDKKQNVY<br>ALFYKLDIVPINNNSEYRLINCNTSVCKQACPKVSFDPIPIHYCAPAGYAILRCNDKNFNGTGPCKNVSSV<br>QCTHGIKPVVSTQLLLNGSLAEDEIIIRSENLTDNVKTIIVHLNKSVEINCTRPSNNTRTSVRIGPGQVFY<br>RTGDIIGDIRKAYCNISRTKWHETLKQVATKLREHFNKTIIFQPPSGGDIEITMHHFNCRGEFFYCNTTKL<br>FNSTWGENTTMEGHNDTIVLPCRIKQIVNMWQGVGQCMYAPPIRGSINCVSNITGILLTRDGGTNMSNETF<br>RPGGGNIKDNWRSELYKYKVVEIEPLGVAPTRCKRRVVGRRRRRRAVGIGAVSLGFLGAAGSTMGAASMTL<br>TVQARNLLSGIVQQQSNLLRAPEPQQHLLKDTHWGIKQLQARVLAVEHYLRDQQLLGIWGCSGKLICCTNV<br>PWNSSWSNRNLSEIWDNMTWLQWDKEISNYTQIIYGLLEESQNQQEKNEQDLLALD |
| CNE8 N276A SOSIP | AENLWVTVYYGVPVWRDADTTLFCASDAKAYDTEVHNVWATHACVPTDPNPQEIHLENVTENFNMWKNKMA<br>EQMQEDVISLWDESLKPCVQLTPLCVTLNCTNANLNATVNASTTIGNITDEVRNCSFNTTTELRDKKQNVY<br>ALFYKLDIVPINNNSEYRLINCNTSVCKQACPKVSFDPIPIHYCAPAGYAILRCNDKNFNGTGPCKNVSSV<br>QCTHGIKPVVSTQLLLNGSLAEDEIIIRSEALTDNVKTIIVHLNKSVEINCTRPSNNTRTSVRIGPGQVFY<br>RTGDIIGDIRKAYCNISRTKWHETLKQVATKLREHFNKTIIFQPPSGGDIEITMHHFNCRGEFFYCNTTKL<br>FNSTWGENTTMEGHNDTIVLPCRIKQIVNMWQGVGQCMYAPPIRGSINCVSNITGILLTRDGGTNMSNETF<br>RPGGGNIKDNWRSELYKYKVVEIEPLGVAPTRCKRRVVGRRRRRRAVGIGAVSLGFLGAAGSTMGAASMTL<br>TVQARNLLSGIVQQQSNLLRAPEPQQHLLKDTHWGIKQLQARVLAVEHYLRDQQLLGIWGCSGKLICCTNV<br>PWNSSWSNRNLSEIWDNMTWLQWDKEISNYTQIIYGLLEESQNQQEKNEQDLLALD |
| CNE20 SOSIP | NLWVTVYYGVPVWKEATTTLFCASDAKAYDTEVHNVWATHACVPTDPNPHELVLENVTENFNMWKNEMVNQ<br>MHEDVISLWDQSLKPCVKLTPLCVTLECGNITTRKESMTEMKNCSFNATTVVKDRKQTVYALFYKLDIVPL<br>SGKNSSGYYRLINCNTSACTQACPKVNFDPIPIHYCTPAGYAILKCNDKTFNGTGPCHNVSTVQCTHGIKP<br>VISTQLLLNGSLAEGEIVIRSENLTNNAKIIIVHLNQTVEIVCTRPGNNTRKSIRIGPGQTFYATGEIIGN<br>IRQAHCNISENQWHKTLQNVSKKLAEHFQNKTITFASSSGGDLEITTHSFNCRGEFFYCNTSGLFNGTYMS<br>NNTEGNSSSIITIPCRIKQIINMWQEVGRCIYAPPIEGNITCKSNITGLLLERDGGTESNDTEIFRPGGGD<br>MRNNWRSELYKYKVVEIKPLGVAPTRCKRRVVGRRRRRRAVGIGAVSLGFLGAAGSTMGAASMTLTVQARN<br>LLSGIVQQQSNLLRAPEPQQHLLKDTHWGIKQLQARVLAVEHYLRDQQLLGIWGCSGKLICCTNVPWNSSW<br>SNRNLSEIWDNMTWLQWDKEISNYTQIIYGLLEESQNQQEKNEQDLLALD |
| CNE20 N276A SOSIP | NLWVTVYYGVPVWKEATTTLFCASDAKAYDTEVHNVWATHACVPTDPNPHELVLENVTENFNMWKNEMVNQ<br>MHEDVISLWDQSLKPCVKLTPLCVTLECGNITTRKESMTEMKNCSFNATTVVKDRKQTVYALFYKLDIVPL<br>SGKNSSGYYRLINCNTSACTQACPKVNFDPIPIHYCTPAGYAILKCNDKTFNGTGPCHNVSTVQCTHGIKP<br>VISTQLLLNGSLAEGEIVIRSEALTNNAKIIIVHLNQTVEIVCTRPGNNTRKSIRIGPGQTFYATGEIIGN<br>IRQAHCNISENQWHKTLQNVSKKLAEHFQNKTITFASSSGGDLEITTHSFNCRGEFFYCNTSGLFNGTYMS<br>NNTEGNSSSIITIPCRIKQIINMWQEVGRCIYAPPIEGNITCKSNITGLLLERDGGTESNDTEIFRPGGGD<br>MRNNWRSELYKYKVVEIKPLGVAPTRCKRRVVGRRRRRRAVGIGAVSLGFLGAAGSTMGAASMTLTVQARN<br>LLSGIVQQQSNLLRAPEPQQHLLKDTHWGIKQLQARVLAVEHYLRDQQLLGIWGCSGKLICCTNVPWNSSW<br>SNRNLSEIWDNMTWLQWDKEISNYTQIIYGLLEESQNQQEKNEQDLLALD |
| IOMA HC Fab | EVQLVESGAQVKKPGASVTVSCTASGYKFTGYHMHWVRQAPGRGLEWMGWINPFRGAVKYPQNFRGRVSMT<br>RDTSMEIFYMELSRLTSDDTAVYYCAREMFDSSADWSPWRGMVAWGQGTLVTVSSASTKGPSVFPLAPSSK<br>STSGGTAALGCLVKDYFPEPVTVSWNSGALTSGVHTFPAVLQSSGLYSLSSVVTVPSSSLGTQTYICNVNH<br>KPSNTKVDKRVEPKSCDKT |

| | |
|---|---|
| IOMA HC | EVQLVESGAQVKKPGASVTVSCTASGYKFTGYHMHWVRQAPGRGLEWMGWINPFRGAVKYPQNFRGRVSMTRDTSMEIFYMELSRLTSDDTAVYYCAREMFDSSADWSPWRGMVAWGQGTLVTVSSASTKGPSVFPLAPSSKSTSGGTAALGCLVKDYFPEPVTVSWNSGALTSGVHTFPAVLQSSGLYSLSSVVTVPSSSLGTQTYICNVNHKPSNTKVDKRVEPKSCDKTHTCPPCPAPELLGGPSVFLFPPKPKDTLMISRTPEVTCVVVDVSHEDPEVKFNWYVDGVEVHNAKTKPREEQYNSTYRVVSVLTVLHQDWLNGKEYKCKVSNKALPAPIEKTISKAKGQPREPQVYTLPPSREEMTKNQVSLTCLVKGFYPSDIAVEWESNGQPENNYKTTPPVLDSDGSFFLYSKLTVDKSRWQQGNVFSCSVMHEALHNHYTQKSLSLSPGK |
| IOMA LC | QSALTQPASVSGSPGQSITISCAGSSRDVGGFDLVSWYQQHPGKAPKLIIYEVNKRPSGISSRFSASKSGNTASLTISGLQEEDEAHYYCYSYADGVAFGGGTKLTVLGQPKAAPSVTLFPPSSEELQANKATLVCLISDFYPGAVTVAWKADSSPVKAGVETTTPSKQSNNKYAASSYLSLTPEQWKSHRSYSCQVTHEGSTVEKTVAPTECS |
| IOMA iGL HC Fab | QVQLVQSGAEVKKPGASVKVSCKASGYTFTGYYMHWVRQAPGQGLEWMGWINPNSGGTNYAQKFQGRVTMTRDTSISTAYMELSRLRSDDTAVYYCARDFTSSYDSSGYYHEGYWGQGTLVTVSSASTKGPSVFPLAPSSKSTSGGTAALGCLVKDYFPEPVTVSWNSGALTSGVHTFPAVLQSSGLYSLSSVVTVPSSSLGTQTYICNVNHKPSNTKVDKRVEPKSCDKT |
| IOMA iGL HC | QVQLVQSGAEVKKPGASVKVSCKASGYTFTGYYMHWVRQAPGQGLEWMGWINPNSGGTNYAQKFQGRVTMTRDTSISTAYMELSRLRSDDTAVYYCARDFTSSYDSSGYYHEGYWGQGTLVTVSSASTKGPSVFPLAPSSKSTSGGTAALGCLVKDYFPEPVTVSWNSGALTSGVHTFPAVLQSSGLYSLSSVVTVPSSSLGTQTYICNVNHKPSNTKVDKRVEPKSCDKTHTCPPCPAPELLGGPSVFLFPPKPKDTLMISRTPEVTCVVVDVSHEDPEVKFNWYVDGVEVHNAKTKPREEQYNSTYRVVSVLTVLHQDWLNGKEYKCKVSNKALPAPIEKTISKAKGQPREPQVYTLPPSREEMTKNQVSLTCLVKGFYPSDIAVEWESNGQPENNYKTTPPVLDSDGSFFLYSKLTVDKSRWQQGNVFSCSVMHEALHNHYTQKSLSLSPGK |
| IOMA iGL LC | QSALTQPASVSGSPGQSITISCTGTSSDVGSYNLVSWYQQHPGKAPKLMIYEVSKRPSGVSNRFSGSKSGNTASLTISGLQAEDEADYYCCSYAGSVAFGGGTKLTVLGQPKAAPSVTLFPPSSEELQANKATLVCLISDFYPGAVTVAWKADSSPVKAGVETTTPSKQSNNKYAASSYLSLTPEQWKSHRSYSCQVTHEGSTVEKTVAPTECS |
| VRC01 iGL HC | QVQLVQSGAEVKKPGASVKVSCKASGYTFTGYYMHWVRQAPGQGLEWMGWINPNSGGTNYAQKFQGRVTMTRDTSISTAYMELSRLRSDDTAVYYCARGKNSDYNWDFQHWGQGTLVTVSSASTKGPSVFPLAPSSKSTSGGTAALGCLVKDYFPEPVTVSWNSGALTSGVHTFPAVLQSSGLYSLSSVVTVPSSSLGTQTYICNVNHKPSNTKVDKRVEPKSCDKTHTCPPCPAPELLGGPSVFLFPPKPKDTLMISRTPEVTCVVVDVSHEDPEVKFNWYVDGVEVHNAKTKPREEQYNSTYRVVSVLTVLHQDWLNGKEYKCKVSNKALPAPIEKTISKAKGQPREPQVYTLPPSREEMTKNQVSLTCLVKGFYPSDIAVEWESNGQPENNYKTTPPVLDSDGSFFLYSKLTVDKSRWQQGNVFSCSVMHEALHNHYTQKSLSLSPGK |
| VRC01 iGL LC | EIVLTQSPATLSLSPGERATLSCRASQSVSSYLAWYQQKPGQAPRLLIYDASNRATGIPARFSGSGSGTDFTLTISSLEPEDFAVYYCQQYEFFGQGTKLEIKRTVAAPSVFIFPPSDEQLKSGTASVVCLLNNFYPREAKVQWKVDNALQSGNSQESVTEQDSKDSTYSLSSTLTLSKADYEKHKVYACEVTHQGLSSPVTKSFNRGEC |
| 3BNC60 iGL HC | QVQLVQSGAEVKKPGASVKVSCKASGYTFTGYYMHWVRQAPGQGLEWMGWINPNSGGTNYAQKFQGRVTMTRDTSISTAYMELSRLRSDDTAVYYCARERSDFWDFDLWGRGTLVTVSSASTKGPSVFPLAPSSKSTSGGTAALGCLVKDYFPEPVTVSWNSGALTSGVHTFPAVLQSSGLYSLSSVVTVPSSSLGTQTYICNVNHKPSNTKVDKRVEPKSCDKTHTCPPCPAPELLGGPSVFLFPPKPKDTLMISRTPEVTCVVVDVSHEDPEVKFNWYVDGVEVHNAKTKPREEQYNSTYRVVSVLTVLHQDWLNGKEYKCKVSNKALPAPIEKTISKAKGQPREPQVYTLPPSREEMTKNQVSLTCLVKGFYPSDIAVEWESNGQPENNYKTTPPVLDSDGSFFLYSKLTVDKSRWQQGNVFSCSVMHEALHNHYTQKSLSLSPGK |
| 3BNC60 iGL LC | DIQMTQSPSSLSASVGDRVTITCQASQDISNYLNWYQQKPGKAPKLLIYDASNLETGVPSRFSGSGSGTDFTFTISSLQPEDIATYYCQQYEFIGPGTKVDIKRTVAAPSVFIFPPSDEQLKSGTASVVCLLNNFYPREAKVQWKVDNALQSGNSQESVTEQDSKDSTYSLSSTLTLSKADYEKHKVYACEVTHQGLSSPVTKSFNRGEC |
| BG24 iGL HC | QVQLVQSGAEVKKPGASVKVSCKASGYTFTGYYMHWVRQAPGQGLEWMGWINPNSGGTNYAQKFQGRVTMTRDTSISTAYMELSRLRSDDTAVYYCATQLELDSSAGYAFDIWGQGTMVTVSSASTKGPSVFPLAPSSKSTSGGTAALGCLVKDYFPEPVTVSWNSGALTSGVHTFPAVLQSSGLYSLSSVVTVPSSSLGTQTYICNVNHKPSNTKVDKRVEPKSCDKTHTCPPCPAPELLGGPSVFLFPPKPKDTLMISRTPEVTCVVVDVSHEDPEVKFNWYVDGVEVHNAKTKPREEQYNSTYRVVSVLTVLHQDWLNGKEYKCKVSNKALPAPIEKTISKAKGQPREPQVYTLPPSREEMTKNQVSLTCLVKGFYPSDIAVEWESNGQPENNYKTTPPVLDSDGSFFLYSKLTVDKSRWQQGNVFSCSVMHEALHNHYTQKSLSLSPGK |

| | |
|---|---|
| BG24 iGL LC | QSALTQPRSVSGSPGQSVTISCTGTSSDVGGYNYVSWYQQHPGKAPKLMIYDVSKRPSGVPDRFSGSKSGN TASLTISGLQAEDEADYYCSSYEYFGGGTKLTVLSQPKAAPSVTLFPPSSEELQANKATLVCLISDFYPGA VTVAWKADSSPVKAGVETTTPSKQSNNKYAASSYLSLTPEQWKSHRSYSCQVTHEGSTVEKTVAPTECS |

**Table S2: X-ray data collection for IOMA iGL Fab crystals**

| | |
|---|---|
| Space group | P $2_1$ $2_1$ $2_1$ |
| Cell dimensions | |
| *a*, *b*, *c* (Å) | 57.7, 66.7, 166.3 |
| *α*, *β*, *γ* (°) | 90, 90, 90 |
| Resolution (Å) | 38.6–2.07 (2.15–2.07)[a] |
| $R_{merge}$ | 0.08 (0.58) |
| $R_{pim}$ | 0.05 (0.36) |
| $I/\sigma(I)$ | 9.7 (2.5) |
| $CC_{1/2}$ | 0.99 (0.92) |
| Completeness (%) | 99 (99) |
| Redundancy | 6.3 (6.6) |
| **Refinement** | |
| Resolution (Å) | 38.6–2.07 |
| No. reflections | 39,372 |
| $R_{work}$ / $R_{free}$ | 0.224 / 0.257 |
| No. atoms | |
| Protein | 3,241 |
| Ligand/ion | N/A |
| *B* factors (Å$^2$) | |
| Protein | 46.7 |
| Ligand/ion | N/A |
| R.m.s. deviations | |
| Bond lengths (Å) | 0.008 |
| Bond angles (°) | 1.00 |

[a] Values in parentheses are for the highest-resolution shell.

# Table S3: Serum neutralization data for IOMA iGL transgenic mice

| | | | ES30 | | ES32 | | ES34 | | ES37 | | ET33 | | ET34 | |
|---|---|---|---|---|---|---|---|---|---|---|---|---|---|---|
| | | | B3 (week 18) | | B3 (week 18) | | B3 (week 18) | | B3 (week 18) | | B3 (week 18) | | B3 (week 18) | |
| Virus | Clade | Tier | $ID_{50}$ | % 1:100 | $ID_{50}$ | % 1:100 | $ID_{50}$ | % 1:100 | $ID_{50}$ | % 1:100 | $ID_{50}$ | % 1:100 | $ID_{50}$ | % 1:100 |
| 426c | C | 2 | – | – | – | – | – | – | – | – | <100 | 0 | <100 | 0 |
| 25710 | B | 2 | – | – | – | – | – | – | – | – | <100 | 0 | <100 | 0 |
| CNE8 | AE | 1 | <100 | 0 | <100 | 0 | <100 | 0 | <100 | 0 | <100 | 0 | 104 | 54 |
| CNE8 N276A | AE | 1 | 463 | 71 | <100 | 0 | <100 | 0 | <100 | 0 | <100 | 0 | <100 | 40 |
| CNE20 | BC | 2 | <100 | 49 | <100 | 0 | <100 | 0 | <100 | 0 | <100 | 0 | <100 | 0 |
| CNE20 N276A | BC | 2 | 14,922 | 95 | <100 | 0 | <100 | 0 | <100 | 0 | <100 | 0 | <100 | 36 |
| JRCSF | B | 2 | 136 | 65 | <100 | 0 | <100 | 0 | <100 | 0 | <100 | 0 | <100 | 0 |
| Q23.17 | A | 1 | 100 | 51 | <100 | 0 | <100 | 0 | <100 | 0 | <100 | 0 | <100 | 26 |
| YU2 | B | 2 | 571 | 86 | <100 | 0 | <100 | 0 | <100 | 0 | <100 | 0 | 112 | 56 |
| BG505 T332N | A | 2 | – | – | – | – | – | – | – | – | <100 | 0 | <100 | 0 |
| 6535.5 | B | 1 | – | – | – | – | – | – | – | – | <100 | 0 | 104 | 53 |
| 3415_V1_C1 | A | 2 | – | – | – | – | – | – | – | – | 154 | 53 | 102 | 59 |
| CAAN5342.A2 | B | 2 | – | – | – | – | – | – | – | – | <100 | 0 | <100 | 41 |
| PVO.4 | B | 3 | 113 | 52 | <100 | 0 | <100 | 0 | <100 | 0 | <100 | 22 | <100 | 57 |
| Q842.D12 | A | 2 | – | – | – | – | – | – | – | – | <100 | 0 | <100 | 0 |
| RHPA4259.7 | B | 2 | – | – | – | – | – | – | – | – | <100 | 0 | <100 | 43 |
| WITO4160.33 | B | 2 | <100 | 0 | <100 | 0 | <100 | 0 | <100 | 0 | <100 | 0 | <100 | 45 |
| ZM214M.PL15 | C | 2 | – | – | – | – | – | – | – | – | <100 | 0 | <100 | 0 |
| MuLV | | | <100 | 0 | <100 | 0 | <100 | 0 | <100 | 0 | <100 | 0 | 162 | 65 |

| | | | HP1 | | HP2 | | HP3 | | HP4 | | HP6 | | HP7 | | HQ4 | |
|---|---|---|---|---|---|---|---|---|---|---|---|---|---|---|---|---|
| | | | B4 (week 23) | | B4 (week 23) | | B4 (week 23) | | B4 (week 23) | | B4 (week 23) | | B4 (week 23) | | B4 (week 23) | |
| Virus | Clade | Tier | $ID_{50}$ | % 1:100 | $ID_{50}$ | % 1:100 | $ID_{50}$ | % 1:100 | $ID_{50}$ | % 1:100 | $ID_{50}$ | % 1:100 | $ID_{50}$ | % 1:100 | $ID_{50}$ | % 1:100 |
| **426c** | C | 2 | <100 | 0 | <100 | 0 | <100 | 0 | <100 | 0 | – | – | <100 | 0 | <100 | 0 |
| **25710** | B | 2 | <100 | 0 | <100 | 0 | <100 | 0 | <100 | 0 | – | – | <100 | 0 | <100 | 0 |
| **CNE8** | AE | 1 | <100 | 0 | <100 | 0 | <100 | 0 | <100 | 0 | – | – | <100 | 0 | <100 | 0 |
| **CNE8 N276A** | AE | 1 | <100 | 26 | <100 | 0 | <100 | 0 | <100 | 12 | – | – | <100 | 0 | <100 | 0 |
| **CNE20** | BC | 2 | <100 | 23 | <100 | 0 | <100 | 40 | <100 | 0 | – | – | <100 | 0 | <100 | 0 |
| **CNE20 N276A** | BC | 2 | 338 | 83 | 610 | 79 | 903 | 88 | <100 | 16 | – | – | 2,017 | 98 | <100 | 0 |
| **JRCSF** | B | 2 | <100 | 0 | <100 | 0 | <100 | 0 | <100 | 0 | – | – | <100 | 0 | <100 | 0 |
| **Q23.17** | A | 1 | 120 | 57 | <100 | 30 | <100 | 28 | <100 | 0 | – | – | <100 | 0 | <100 | 0 |
| **YU2** | B | 2 | <100 | 0 | <100 | 0 | <100 | 0 | <100 | 0 | – | – | <100 | 0 | <100 | 0 |
| **BG505 T332N** | A | 2 | <100 | 0 | <100 | 0 | <100 | 0 | <100 | 0 | – | – | <100 | 0 | <100 | 0 |
| **6535.5** | B | 1 | 165 | 67 | <100 | 0 | <100 | 0 | <100 | 0 | – | – | <100 | 0 | <100 | 0 |
| **3415_V1_C1** | A | 2 | <100 | 0 | <100 | 40 | <100 | 0 | <100 | 0 | – | – | <100 | 0 | 108 | 50 |
| **CAAN5342.A2** | B | 2 | <100 | 0 | <100 | 0 | <100 | 0 | <100 | 0 | – | – | <100 | 0 | <100 | 0 |
| **PVO.4** | B | 3 | <100 | 43 | <100 | 45 | <100 | 12 | <100 | 35 | – | – | 115 | 50 | <100 | 0 |
| **Q842.D12** | A | 2 | <100 | 0 | <100 | 0 | <100 | 0 | <100 | 0 | – | – | <100 | 0 | <100 | 0 |
| **RHPA4259.7** | B | 2 | <100 | 0 | <100 | 0 | <100 | 0 | <100 | 0 | – | – | <100 | 0 | <100 | 0 |
| **WITO4160.33** | B | 2 | <100 | 28 | 145 | 53 | <100 | 0 | <100 | 48 | – | – | <100 | 40 | <100 | 0 |
| **ZM214 M.PL15** | C | 2 | <100 | 0 | <100 | 0 | <100 | 0 | <100 | 0 | – | – | <100 | 0 | <100 | 0 |
| **MuLV** | | | <100 | 0 | <100 | 0 | <100 | 0 | <100 | 0 | – | – | <100 | 0 | <100 | 0 |

**Table S4: Mutational analysis of antibodies isolated from IOMA iGL transgenic mice.**

| VH1-2*02 amino acid | VH Position | amino acid substitution | Random Frequency | # of IGT2-induced mAbs with this SHM | IGT2 Frequency | IOMA Substitution | Critical Interaction | VRC01 Substitution |
|---|---|---|---|---|---|---|---|---|
| Q | 1 | | – | 67 | 100.0 | E | | |
| | | E | 0.0 | 0 | 0.0 | | | |
| V | 2 | | | 67 | 100.0 | V | | |
| Q | 3 | | | 67 | 100.0 | Q | | |
| L | 4 | | | 67 | 100.0 | L | | |
| V | 5 | | | 67 | 100.0 | V | | |
| Q | 6 | | | 67 | 100.0 | E | | |
| | | E | 0.1 | 0 | 0.0 | | | |
| S | 7 | | | 67 | 100.0 | S | | |
| G | 8 | | | 67 | 100.0 | G | | |
| A | 9 | | | 67 | 100.0 | A | | G |
| E | 10 | | | 67 | 100.0 | Q | | Q |
| | | Q | 0.2 | 0 | 0.0 | | | |
| V | 11 | | | 66 | 98.5 | V | | M |
| | | M | 3.3 | 1 | 1.5 | | | |
| K | 12 | | | 56 | 83.6 | K | | |
| | | R | 6.9 | 11 | 16.4 | | | |
| K | 13 | | | 67 | 100.0 | K | | |
| P | 14 | | | 67 | 100.0 | P | | |
| G | 15 | | | 67 | 100.0 | G | | E |
| A | 16 | | | 67 | 100.0 | A | | |
| S | 17 | | | 67 | 100.0 | S | | |
| V | 18 | | | 67 | 100.0 | V | | M |
| K | 19 | | | 22 | 32.8 | T | **YES** | R |
| | | T | 2.3 | 31 | 46.3 | | | |
| | | R | 9.6 | 14 | 20.9 | | | |
| V | 20 | | | 67 | 100.0 | V | | I |
| S | 21 | | | 67 | 100.0 | S | | |
| C | 22 | | | 67 | 100.0 | C | | |
| K | 23 | | | 55 | 82.1 | T | | R |
| | | A | 0.2 | 1 | 1.5 | | | |
| | | T | 2.3 | 0 | 0.0 | | | |
| | | E | 3.3 | 5 | 7.5 | | | |
| | | R | 4.6 | 6 | 9.0 | | | |
| A | 24 | | | 61 | 91.0 | A | | |
| | | T | 13.5 | 6 | 9.0 | | | |
| S | 25 | | | 67 | 100.0 | S | | |
| G | 26 | | | 67 | 100.0 | G | | |

| | | | | | | | | |
|---|---|---|---|---|---|---|---|---|
| Y | 27 | | | 67 | 100.0 | Y | | |
| T | 28 | | | 65 | 97.0 | K | | E |
| | | K | 0.6 | 0 | 0.0 | | | |
| | | N | 2.0 | 2 | 3.0 | | | |
| F | 29 | | | 66 | 98.5 | F | | |
| | | L | 3.8 | 1 | 1.5 | | | |
| T | 30 | | | 58 | 86.6 | T | | I |
| | | A | 1.7 | 1 | 1.5 | | | |
| | | I | 7.2 | 8 | 11.9 | | | |
| G | 31 | | | 32 | 47.8 | G | | D |
| | | E | 0.8 | 1 | 1.5 | | | |
| | | A | 11.7 | 6 | 9.0 | | | |
| | | D | 34.3 | 28 | 41.8 | | | |
| Y | 32 | | | 65 | 97.0 | Y | | C |
| | | H | 8.5 | 2 | 3.0 | | | |
| Y | 33 | | | 27 | 40.3 | H | **YES** | T |
| | | E | 0.1 | 14 | 20.9 | | | |
| | | D | 0.6 | 9 | 13.4 | | | |
| | | S | 1.9 | 2 | 3.0 | | | |
| | | H | 4.5 | 8 | 11.9 | | | |
| | | F | 8.4 | 7 | 10.4 | | | |
| M | 34 | | | 33 | 49.3 | M | | L |
| | | L | 13.8 | 7 | 10.4 | | | |
| | | I | 48.4 | 27 | 40.3 | | | |
| H | 35 | | | 61 | 91.0 | H | | N |
| | | Q | 2.2 | 1 | 1.5 | | | |
| | | Y | 3.5 | 5 | 7.5 | | | |
| W | 36 | | | 67 | 100.0 | W | | |
| V | 37 | | | 67 | 100.0 | V | | I |
| R | 38 | | | 67 | 100.0 | R | | |
| Q | 39 | | | 66 | 98.5 | Q | | L |
| | | R | 1.1 | 1 | 1.5 | | | |
| A | 40 | | | 65 | 97.0 | A | | |
| | | V | 2.0 | 2 | 3.0 | | | |
| P | 41 | | | 67 | 100.0 | P | | |
| G | 42 | | | 67 | 100.0 | G | | |
| Q | 43 | | | 66 | 98.5 | R | | K |
| | | R | 1.5 | 1 | 1.5 | | | |
| G | 44 | | | 67 | 100.0 | G | | R |
| L | 45 | | | 64 | 95.5 | L | | P |
| | | F | 1.9 | 3 | 4.5 | | | |
| E | 46 | | | 66 | 98.5 | E | | |

| | | | | | | | | |
|---|---|---|---|---|---|---|---|---|
| | | D | 0.5 | 1 | 1.5 | | | |
| W | 47 | | | 67 | 100.0 | W | | |
| M | 48 | | | 62 | 92.5 | M | | |
| | | L | 4.9 | 4 | 6.0 | | | |
| | | V | 6.4 | 1 | 1.5 | | | |
| G | 49 | | | 67 | 100.0 | G | | |
| W | 50 | | | 52 | 77.6 | W | | |
| | | R | 0.0 | 14 | 20.9 | | | |
| | | L | 0.8 | 1 | 1.5 | | | |
| I | 51 | | | 66 | 98.5 | I | | L |
| | | S | 0.5 | 1 | 1.5 | | | |
| N | 52 | | | 58 | 86.6 | N | | K |
| | | H | 2.2 | 3 | 4.5 | | | |
| | | S | 4.4 | 6 | 9.0 | | | |
| P | (52A) | | | 67 | 100.0 | P | | |
| N | 53 | | | 15 | 22.4 | F | **YES** | R |
| | | F | 0.1 | 30 | 44.8 | | | |
| | | E | 1.0 | 2 | 3.0 | | | |
| | | T | 1.4 | 5 | 7.5 | | | |
| | | R | 1.8 | 7 | 10.4 | | | |
| | | Y | 3.5 | 5 | 7.5 | | | |
| | | D | 8.0 | 1 | 1.5 | | | |
| | | K | 13.5 | 2 | 3.0 | | | |
| S | 54 | | | 12 | 17.9 | R | **YES** | G |
| | | F | 0.2 | 7 | 10.4 | | | |
| | | R | 2.7 | 45 | 67.2 | | | |
| | | N | 11.9 | 1 | 1.5 | | | |
| | | T | 14.8 | 2 | 3.0 | | | |
| G | 55 | | | 67 | 100.0 | G | | |
| G | 56 | | | 31 | 46.3 | A | **YES** | A |
| | | R | 0.9 | 2 | 3.0 | | | |
| | | N | 0.9 | 8 | 11.9 | | | |
| | | V | 6.3 | 5 | 7.5 | | | |
| | | A | 11.3 | 20 | 29.9 | | | |
| | | D | 22.4 | 1 | 1.5 | | | |
| T | 57 | | | 21 | 31.3 | V | **YES** | V |
| | | V | 0.4 | 21 | 31.3 | | | |
| | | R | 0.9 | 3 | 4.5 | | | |
| | | P | 1.3 | 2 | 3.0 | | | |
| | | I | 1.4 | 18 | 26.9 | | | |
| | | S | 1.9 | 2 | 3.0 | | | |
| N | 58 | | | 18 | 26.9 | K | **YES** | |

| | | | | | | | | |
|---|---|---|---|---|---|---|---|---|
| | | G | 0.7 | 10 | 14.9 | | | |
| | | E | 1.4 | 15 | 22.4 | | | |
| | | D | 7.2 | 8 | 11.9 | | | |
| | | K | 16.5 | 16 | 23.9 | | | |
| Y | 59 | | | 52 | 77.6 | Y | | |
| | | C | 0.7 | 2 | 3.0 | | | |
| | | S | 5.6 | 13 | 19.4 | | | |
| A | 60 | | | 42 | 62.7 | P | | |
| | | R | 0.1 | 3 | 4.5 | | | |
| | | E | 1.6 | 3 | 4.5 | | | |
| | | T | 1.9 | 6 | 9.0 | | | |
| | | P | 2.1 | 0 | 0.0 | | | |
| | | V | 2.2 | 8 | 11.9 | | | |
| | | S | 3.0 | 5 | 7.5 | | | |
| Q | 61 | | | 56 | 83.6 | Q | | R |
| | | R | 2.8 | 3 | 4.5 | | | |
| | | E | 4.3 | 8 | 11.9 | | | |
| K | 62 | | | 64 | 95.5 | N | | P |
| | | R | 7.5 | 1 | 1.5 | | | |
| | | N | 9.4 | 2 | 3.0 | | | |
| F | 63 | | | 66 | 98.5 | F | | L |
| | | L | 2.4 | 1 | 1.5 | | | |
| Q | 64 | | | 56 | 83.6 | R | | |
| | | R | 4.8 | 11 | 16.4 | | | |
| G | 65 | | | 67 | 100.0 | G | | |
| R | 66 | | | 67 | 100.0 | R | | |
| V | 67 | | | 66 | 98.5 | V | | |
| | | L | 2.3 | 1 | 1.5 | | | |
| T | 68 | | | 64 | 95.5 | S | | |
| | | I | 2.4 | 2 | 3.0 | | | |
| | | S | 4.4 | 1 | 1.5 | | | |
| M | 69 | | | 64 | 95.5 | M | | |
| | | L | 12.3 | 3 | 4.5 | | | |
| T | 70 | | | 67 | 100.0 | T | | |
| R | 71 | | | 67 | 100.0 | R | **YES** | |
| D | 72 | | | 67 | 100.0 | D | | |
| T | 73 | | | 66 | 98.5 | T | | V |
| | | P | 0.8 | 1 | 1.5 | | | |
| S | 74 | | | 66 | 98.5 | S | | Y |
| | | T | 0.7 | 1 | 1.5 | | | |
| I | 75 | | | 67 | 100.0 | M | | S |
| | | M | 1.5 | 0 | 0.0 | | | |

| | | | | | | | | |
|---|---|---|---|---|---|---|---|---|
| S | 76 | | | 37 | 55.2 | E | | D |
| | | E | 0.1 | 0 | 0.0 | | | |
| | | I | 0.3 | 1 | 1.5 | | | |
| | | K | 0.6 | 1 | 1.5 | | | |
| | | R | 3.1 | 1 | 1.5 | | | |
| | | T | 13.3 | 20 | 29.9 | | | |
| | | N | 18.0 | 7 | 10.4 | | | |
| T | 77 | | | 64 | 95.5 | I | | |
| | | I | 0.6 | 3 | 4.5 | | | |
| A | 78 | | | 60 | 89.6 | F | | |
| | | F | 0.5 | 0 | 0.0 | | | |
| | | T | 2.1 | 1 | 1.5 | | | |
| | | V | 15.3 | 6 | 9.0 | | | |
| Y | 79 | | | 67 | 100.0 | Y | | F |
| M | 80 | | | 64 | 95.5 | M | | L |
| | | L | 9.1 | 3 | 4.5 | | | |
| E | 81 | | | 65 | 97.0 | E | | |
| | | V | 0.4 | 2 | 3.0 | | | |
| L | 82 | | | 66 | 98.5 | L | | |
| | | M | 2.3 | 1 | 1.5 | | | |
| S | (82A) | | | 45 | 67.2 | S | | R |
| | | K | 1.2 | 1 | 1.5 | | | |
| | | R | 8.1 | 2 | 3.0 | | | |
| | | N | 8.9 | 16 | 23.9 | | | |
| | | T | 12.8 | 3 | 4.5 | | | |
| R | (82B) | | | 59 | 88.1 | R | | S |
| | | G | 13.4 | 8 | 11.9 | | | |
| L | (82C) | | | 66 | 98.5 | L | | |
| | | V | 0.9 | 1 | 1.5 | | | |
| R | 83 | | | 60 | 89.6 | T | | T |
| | | N | 0.7 | 1 | 1.5 | | | |
| | | I | 1.7 | 1 | 1.5 | | | |
| | | K | 7.9 | 2 | 3.0 | | | |
| | | T | 29.6 | 3 | 4.5 | | | |
| S | 84 | | | 66 | 98.5 | S | | V |
| | | Y | 2.3 | 1 | 1.5 | | | |
| D | 85 | | | 66 | 98.5 | D | | |
| | | N | 1.0 | 1 | 1.5 | | | |
| D | 86 | | | 67 | 100.0 | D | | |
| T | 87 | | | 67 | 100.0 | T | | |
| A | 88 | | | 67 | 100.0 | A | | |
| V | 89 | | | 60 | 89.6 | V | | |

| | | | | | | | | |
|---|---|---|---|---|---|---|---|---|
| | | R<br>A<br>M<br>I | 0.1<br>0.3<br>4.6<br>10.8 | 1<br>1<br>1<br>4 | 1.5<br>1.5<br>1.5<br>6.0 | | | |
| Y | 90 | | | 67 | 100.0 | Y | | |
| Y | 91 | <br>N<br>F | <br>0.0<br>14.6 | 48<br>2<br>17 | 71.6<br>3.0<br>25.4 | Y | | F |
| C | 92 | | | 67 | 100.0 | C | | |
| A | 93 | <br>T<br>V | <br>3.5<br>5.1 | 65<br>1<br>1 | 97.0<br>1.5<br>1.5 | A | | T |
| R | 94 | | | 67 | 100.0 | R | | |

| VL2-23*02 amino acid | VL Position | amino acid substitution | Random Frequency | # of IGT2 induced mAbs with this SHM | IGT2 Frequency | IOMA Substitution | Critical Interaction | VK3-20*01 Residue | VL Position | VRC01 Substitution | Random Frequency | Critical Interaction |
|---|---|---|---|---|---|---|---|---|---|---|---|---|
| Q | 1 | | | 67 | 100.0 | Q | | Q | 1 | Q | 100.0 | |
| S | 2 | | | 66 | 98.5 | S | | S | 2 | S | 98.5 | |
| | | F | 0.1 | 1 | 1.5 | | | | | | 1.5 | |
| A | 3 | | | 67 | 100.0 | A | | A | 3 | A | 100.0 | |
| L | 4 | | | 67 | 100.0 | L | | L | 4 | L | 100.0 | |
| T | 5 | | | 67 | 100.0 | T | | T | 5 | T | 100.0 | |
| Q | 6 | | | 67 | 100.0 | Q | | Q | 6 | Q | 100.0 | |
| P | 7 | | | 67 | 100.0 | P | | P | 7 | P | 100.0 | |
| A | 8 | | | 67 | 100.0 | A | | A | 8 | A | 100.0 | |
| S | 9 | | | 67 | 100.0 | S | | S | 9 | S | 100.0 | |
| V | 11 | | | 67 | 100.0 | V | | V | 11 | V | 100.0 | |
| S | 12 | | | 66 | 98.5 | S | | S | 12 | S | 98.5 | |
| | | F | 0.1 | 1 | 1.5 | | | | | | 1.5 | |
| G | 13 | | | 67 | 100.0 | G | | G | 13 | G | 100.0 | |
| S | 14 | | | 67 | 100.0 | S | | S | 14 | S | 100.0 | |
| P | 15 | | | 67 | 100.0 | P | | P | 15 | P | 100.0 | |
| G | 16 | | | 62 | 92.5 | G | | G | 16 | G | 92.5 | |
| | | E | 0.1 | 5 | 7.5 | | | | | | 7.5 | |
| Q | 17 | | | 67 | 100.0 | Q | | Q | 17 | Q | 100.0 | |
| S | 18 | | | 67 | 100.0 | S | | S | 18 | S | 100.0 | |
| I | 19 | | | 65 | 97.0 | I | | I | 19 | I | 97.0 | |
| | | S | 0.1 | 1 | 1.5 | | | | | | 1.5 | |
| | | T | 0.1 | 1 | 1.5 | | | | | | 1.5 | |
| T | 20 | | | 67 | 100.0 | T | | T | 20 | T | 100.0 | |
| I | 21 | | | 67 | 100.0 | I | | I | 21 | I | 100.0 | |
| S | 22 | | | 67 | 100.0 | S | | S | 22 | S | 100.0 | |
| C | 23 | | | 67 | 100.0 | C | | C | 23 | C | 100.0 | |
| T | 24 | | | 67 | 100.0 | A | | T | 24 | A | 100.0 | |
| | | A | 2.3 | 0 | 0.0 | | | | | | 0.0 | |
| G | 25 | | | 63 | 94.0 | G | | G | 25 | G | 94.0 | |
| | | V | 0.1 | 4 | 6.0 | | | | | | 6.0 | |
| T | 26 | | | 65 | 97.0 | S | | T | 26 | S | 97.0 | |
| | | P | 0.6 | 1 | 1.5 | | | | | | 1.5 | |
| | | A | 2.6 | 1 | 1.5 | | | | | | 1.5 | |
| | | S | 7.2 | 1 | 1.5 | | | | | | 1.5 | |
| S | 27 | | | 65 | 97.0 | S | | S | 27 | S | 97.0 | |
| | | G | 2.2 | 1 | 1.5 | | | | | | 1.5 | |

| | | | | | | | | | | | | | |
|---|---|---|---|---|---|---|---|---|---|---|---|---|---|
| | | R | 2.5 | 1 | 1.5 | | | | | | 1.5 | |
| S | (27A) | | | 63 | 94.0 | R | **YES** | S | (27A) | R | 94.0 | **YES** |
| | | R | 2.4 | 2 | 3.0 | | | | | | 3.0 | |
| | | N | 6.1 | 2 | 3.0 | | | | | | 3.0 | |
| D | (27B) | | | 67 | 100.0 | D | | D | (27B) | D | 100.0 | |
| V | (27C) | | | 53 | 79.1 | V | | V | (27C) | V | 79.1 | |
| | | F | 1.9 | 1 | 1.5 | | | | | | 1.5 | |
| | | I | 17.0 | 13 | 19.4 | | | | | | 19.4 | |
| G | 28 | | | 67 | 100.0 | G | | G | 28 | G | 100.0 | |
| S | 29 | | | 51 | 76.1 | G | **YES** | S | 29 | G | 76.1 | **YES** |
| | | I | 2.0 | 1 | 1.5 | | | | | | 1.5 | |
| | | R | 3.2 | 4 | 6.0 | | | | | | 6.0 | |
| | | G | 5.3 | 4 | 6.0 | | | | | | 6.0 | |
| | | T | 14.3 | 1 | 1.5 | | | | | | 1.5 | |
| | | N | 14.5 | 6 | 9.0 | | | | | | 9.0 | |
| Y | 30 | | | 56 | 83.6 | F | **YES** | Y | 30 | F | 83.6 | **YES** |
| | | S | 3.0 | 4 | 6.0 | | | | | | 6.0 | |
| | | F | 4.1 | 7 | 10.4 | | | | | | 10.4 | |
| N | 31 | | | 35 | 52.2 | D | **YES** | N | 31 | D | 52.2 | **YES** |
| | | Y | 1.3 | 4 | 6.0 | | | | | | 6.0 | |
| | | D | 10.2 | 28 | 41.8 | | | | | | 41.8 | |
| L | 32 | | | 65 | 97.0 | L | | L | 32 | L | 97.0 | |
| | | F | 8.2 | 2 | 3.0 | | | | | | 3.0 | |
| V | 33 | | | 67 | 100.0 | V | | V | 33 | V | 100.0 | |
| S | 34 | | | 66 | 98.5 | S | | S | 34 | S | 98.5 | |
| | | P | 0.0 | 1 | 1.5 | | | | | | 1.5 | |
| W | 35 | | | 67 | 100.0 | W | | W | 35 | W | 100.0 | |
| Y | 36 | | | 67 | 100.0 | Y | | Y | 36 | Y | 100.0 | |
| Q | 37 | | | 67 | 100.0 | Q | | Q | 37 | Q | 100.0 | |
| Q | 38 | | | 67 | 100.0 | Q | | Q | 38 | Q | 100.0 | |
| H | 39 | | | 67 | 100.0 | H | | H | 39 | H | 100.0 | |
| P | 40 | | | 67 | 100.0 | P | | P | 40 | P | 100.0 | |
| G | 41 | | | 67 | 100.0 | G | | G | 41 | G | 100.0 | |
| K | 42 | | | 66 | 98.5 | K | | K | 42 | K | 98.5 | |
| | | N | 0.7 | 1 | 1.5 | | | | | | 1.5 | |
| A | 43 | | | 61 | 91.0 | A | | A | 43 | A | 91.0 | |
| | | T | 0.8 | 5 | 7.5 | | | | | | 7.5 | |
| | | V | 7.6 | 1 | 1.5 | | | | | | 1.5 | |
| P | 44 | | | 67 | 100.0 | P | | P | 44 | P | 100.0 | |
| K | 45 | | | 67 | 100.0 | K | | K | 45 | K | 100.0 | |
| L | 46 | | | 66 | 98.5 | L | | L | 46 | L | 98.5 | |
| | | F | 2.4 | 1 | 1.5 | | | | | | 1.5 | |

| | | | | | | | | | | | | |
|---|---|---|---|---|---|---|---|---|---|---|---|---|
| M | 47 | | | 63 | 94.0 | I | | M | 47 | I | 94.0 | |
| | | L | 10.7 | 2 | 3.0 | | | | | | 3.0 | |
| | | I | 34.4 | 2 | 3.0 | | | | | | 3.0 | |
| I | 48 | | | 66 | 98.5 | I | | I | 48 | I | 98.5 | |
| | | L | 2.6 | 1 | 1.5 | | | | | | 1.5 | |
| Y | 49 | | | 66 | 98.5 | Y | | Y | 49 | Y | 98.5 | |
| | | H | 1.8 | 1 | 1.5 | | | | | | 1.5 | |
| E | 50 | | | 51 | 76.1 | E | | E | 50 | E | 76.1 | |
| | | K | 0.3 | 4 | 6.0 | | | | | | 6.0 | |
| | | D | 6.5 | 12 | 17.9 | | | | | | 17.9 | |
| V | 51 | | | 67 | 100.0 | V | | V | 51 | V | 100.0 | |
| S | 52 | | | 46 | 68.7 | N | | S | 52 | N | 68.7 | |
| | | I | 3.2 | 1 | 1.5 | | | | | | 1.5 | |
| | | N | 19.8 | 14 | 20.9 | | | | | | 20.9 | |
| | | T | 29.2 | 6 | 9.0 | | | | | | 9.0 | |
| K | 53 | | | 38 | 56.7 | K | | K | 53 | K | 56.7 | |
| | | A | 0.2 | 1 | 1.5 | | | | | | 1.5 | |
| | | R | 3.8 | 23 | 34.3 | | | | | | 34.3 | |
| | | Q | 4.9 | 4 | 6.0 | | | | | | 6.0 | |
| | | E | 7.1 | 1 | 1.5 | | | | | | 1.5 | |
| R | 54 | | | 67 | 100.0 | R | | R | 54 | R | 100.0 | |
| P | 55 | | | 67 | 100.0 | P | | P | 55 | P | 100.0 | |
| S | 56 | | | 67 | 100.0 | S | | S | 56 | S | 100.0 | |
| G | 57 | | | 67 | 100.0 | G | | G | 57 | G | 100.0 | |
| V | 58 | | | 61 | 91.0 | I | | V | 58 | I | 91.0 | |
| | | I | 10.9 | 6 | 9.0 | | | | | | 9.0 | |
| S | 59 | | | 66 | 98.5 | S | | S | 59 | S | 98.5 | |
| | | Y | 0.0 | 1 | 1.5 | | | | | | 1.5 | |
| N | 60 | | | 65 | 97.0 | S | | N | 60 | S | 97.0 | |
| | | S | 7.3 | 1 | 1.5 | | | | | | 1.5 | |
| | | D | 17.3 | 1 | 1.5 | | | | | | 1.5 | |
| R | 61 | | | 67 | 100.0 | R | | R | 61 | R | 100.0 | |
| F | 62 | | | 67 | 100.0 | F | | F | 62 | F | 100.0 | |
| S | 63 | | | 65 | 97.0 | S | | S | 63 | S | 97.0 | |
| | | A | 0.4 | 2 | 3.0 | | | | | | 3.0 | |
| G | 64 | | | 65 | 97.0 | A | | G | 64 | A | 97.0 | |
| | | D | 0.1 | 1 | 1.5 | | | | | | 1.5 | |
| | | A | 5.3 | 1 | 1.5 | | | | | | 1.5 | |
| S | 65 | | | 67 | 100.0 | S | | S | 65 | S | 100.0 | |
| K | 66 | | | 67 | 100.0 | K | | K | 66 | K | 100.0 | |
| S | 67 | | | 65 | 97.0 | S | | S | 67 | S | 97.0 | |
| | | C | 0.0 | 1 | 1.5 | | | | | | 1.5 | |

| | | | | | | | |
|---|---|---|---|---|---|---|---|
| | | A | 0.5 | 1 | 1.5 | | |
| G | 68 | | | 65 | 97.0 | G | |
| | | D | 3.3 | 2 | 3.0 | | |
| N | 69 | | | 66 | 98.5 | N | |
| | | K | 0.8 | 1 | 1.5 | | |
| T | 70 | | | 64 | 95.5 | T | |
| | | M | 0.8 | 2 | 3.0 | | |
| | | S | 0.8 | 1 | 1.5 | | |
| A | 71 | | | 67 | 100.0 | A | |
| S | 72 | | | 67 | 100.0 | S | |
| L | 73 | | | 67 | 100.0 | L | |
| T | 74 | | | 56 | 83.6 | T | |
| | | P | 0.0 | 2 | 3.0 | | |
| | | I | 0.5 | 9 | 13.4 | | |
| I | 75 | | | 67 | 100.0 | I | |
| S | 76 | | | 67 | 100.0 | S | |
| G | 77 | | | 66 | 98.5 | G | |
| | | D | 0.5 | 1 | 1.5 | | |
| L | 78 | | | 32 | 47.8 | L | |
| | | F | 0.0 | 35 | 52.2 | | |
| Q | 79 | | | 65 | 97.0 | Q | |
| | | R | 2.5 | 2 | 3.0 | | |
| A | 80 | | | 59 | 88.1 | E | |
| | | E | 0.1 | 0 | 0.0 | | |
| | | D | 0.3 | 1 | 1.5 | | |
| | | T | 4.9 | 6 | 9.0 | | |
| | | P | 5.1 | 1 | 1.5 | | |
| E | 81 | | | 67 | 100.0 | E | |
| D | 82 | | | 67 | 100.0 | D | |
| E | 83 | | | 57 | 85.1 | E | |
| | | V | 0.0 | 3 | 4.5 | | |
| | | K | 0.1 | 2 | 3.0 | | |
| | | G | 0.2 | 5 | 7.5 | | |
| A | 84 | | | 66 | 98.5 | A | |
| | | G | 4.2 | 1 | 1.5 | | |
| D | 85 | | | 61 | 91.0 | H | |
| | | G | 0.2 | 2 | 3.0 | | |
| | | Y | 0.9 | 1 | 1.5 | | |
| | | N | 2.1 | 1 | 1.5 | | |
| | | H | 2.6 | 0 | 0.0 | | |
| | | E | 4.1 | 2 | 3.0 | | |
| Y | 86 | | | 65 | 97.0 | Y | |

| | | | | |
|---|---|---|---|---|
| | | | 1.5 | |
| G | 68 | G | 97.0 | |
| | | | 3.0 | |
| N | 69 | N | 98.5 | |
| | | | 1.5 | |
| T | 70 | T | 95.5 | |
| | | | 3.0 | |
| | | | 1.5 | |
| A | 71 | A | 100.0 | |
| S | 72 | S | 100.0 | |
| L | 73 | L | 100.0 | |
| T | 74 | T | 83.6 | |
| | | | 3.0 | |
| | | | 13.4 | |
| I | 75 | I | 100.0 | |
| S | 76 | S | 100.0 | |
| G | 77 | G | 98.5 | |
| | | | 1.5 | |
| L | 78 | L | 47.8 | |
| | | | 52.2 | |
| Q | 79 | Q | 97.0 | |
| | | | 3.0 | |
| A | 80 | E | 88.1 | |
| | | | 0.0 | |
| | | | 1.5 | |
| | | | 9.0 | |
| | | | 1.5 | |
| E | 81 | E | 100.0 | |
| D | 82 | D | 100.0 | |
| E | 83 | E | 85.1 | |
| | | | 4.5 | |
| | | | 3.0 | |
| | | | 7.5 | |
| A | 84 | A | 98.5 | |
| | | | 1.5 | |
| D | 85 | H | 91.0 | |
| | | | 3.0 | |
| | | | 1.5 | |
| | | | 1.5 | |
| | | | 0.0 | |
| | | | 3.0 | |
| Y | 86 | Y | 97.0 | |

| | | | | | | | | | | | | |
|---|---|---|---|---|---|---|---|---|---|---|---|---|
| | | N | | 2 | 3.0 | | | | | | 3.0 | |
| Y | 87 | | | 45 | 67.2 | Y | | Y | 87 | Y | 67.2 | |
| | | E | 0.0 | 1 | 1.5 | | | | | | 1.5 | |
| | | D | 0.0 | 5 | 7.5 | | | | | | 7.5 | |
| | | H | 4.3 | 12 | 17.9 | | | | | | 17.9 | |
| | | F | 6.2 | 4 | 6.0 | | | | | | 6.0 | |
| C | 88 | | | 67 | 100.0 | C | | C | 88 | C | 100.0 | |
| C | 89 | | | 57 | 85.1 | Y | | C | 89 | Y | 85.1 | |
| | | W | 0.8 | 1 | 1.5 | | | | | | 1.5 | |
| | | Y | 1.8 | 4 | 6.0 | | | | | | 6.0 | |
| | | S | 10.3 | 5 | 7.5 | | | | | | 7.5 | |
| S | 90 | | | 60 | 89.6 | S | | S | 90 | S | 89.6 | |
| | | L | 0.7 | 6 | 9.0 | | | | | | 9.0 | |
| | | T | 1.0 | 1 | 1.5 | | | | | | 1.5 | |
| Y | 96 | | | 61 | 91.0 | Y | | Y | 96 | Y | 91.0 | |
| | | C | 0.7 | 1 | 1.5 | | | | | | 1.5 | |
| | | S | 2.1 | 3 | 4.5 | | | | | | 4.5 | |
| | | F | 7.4 | 2 | 3.0 | | | | | | 3.0 | |
| A | 97 | | | 48 | 71.6 | A | | A | 97 | A | 71.6 | |
| | | E | 1.4 | 1 | 1.5 | | | | | | 1.5 | |
| | | T | 5.8 | 3 | 4.5 | | | | | | 4.5 | |
| | | G | 7.2 | 9 | 13.4 | | | | | | 13.4 | |
| | | V | 8.8 | 6 | 9.0 | | | | | | 9.0 | |

## Table S5: Serum neutralization in wildtype mice

| | | | M1 | | M3 | | M4 | | M5 | | M13 | | M14 | | M15 | | M21 | |
|---|---|---|---|---|---|---|---|---|---|---|---|---|---|---|---|---|---|---|
| | | | B3 (week 18) | | B3 (week 18) | | B3 (week 18) | | B3 (week 18) | | B3 (week 18) | | B3 (week 18) | | B3 (week 18) | | B4 (week 23) | |
| **Virus** | **Clade** | **Tier** | **$ID_{50}$** | **% 1:100** | **$ID_{50}$** | **% 1:100** | **$ID_{50}$** | **% 1:100** | **$ID_{50}$** | **% 1:100** | **$ID_{50}$** | **% 1:100** | **$ID_{50}$** | **% 1:100** | **$ID_{50}$** | **% 1:100** | **$ID_{50}$** | **% 1:100** |
| **426c** | C | 2 | <100 | 0 | <100 | 0 | <100 | 0 | <100 | 0 | <100 | 0 | <100 | 0 | <100 | 0 | <100 | 0 |
| **25710** | B | 2 | <100 | 0 | <100 | 0 | <100 | 0 | <100 | 0 | <100 | 0 | <100 | 0 | <100 | 0 | <100 | 0 |
| **CNE8** | AE | 1 | <100 | 0 | <100 | 0 | <100 | 0 | <100 | 0 | <100 | 0 | <100 | 0 | <100 | 0 | 225 | 56 |
| **CNE8 N276A** | AE | 1 | <100 | 0 | <100 | 0 | <100 | 0 | <100 | 0 | <100 | 0 | <100 | 0 | <100 | 0 | <100 | 0 |
| **CNE20** | BC | 2 | <100 | 0 | <100 | 0 | <100 | 0 | <100 | 0 | <100 | 0 | <100 | 0 | <100 | 40 | <100 | 0 |
| **CNE20 N276A** | BC | 2 | <100 | 0 | <100 | 0 | <100 | 0 | <100 | 0 | <100 | 0 | <100 | 0 | <100 | 0 | <100 | 0 |
| **JRCSF** | B | 2 | <100 | 0 | <100 | 0 | <100 | 0 | <100 | 0 | <100 | 0 | <100 | 0 | <100 | 0 | <100 | 0 |
| **Q23.17** | A | 1 | <100 | 0 | <100 | 0 | <100 | 0 | <100 | 0 | <100 | 0 | <100 | 0 | <100 | 40 | <100 | 0 |
| **YU2** | B | 2 | <100 | 0 | <100 | 0 | <100 | 0 | <100 | 0 | <100 | 0 | <100 | 0 | <100 | 0 | <100 | 0 |
| **BG505 T332N** | A | 2 | <100 | 0 | <100 | 0 | <100 | 0 | <100 | 0 | <100 | 0 | <100 | 0 | <100 | 0 | <100 | 0 |
| **6535.5** | B | 1 | <100 | 0 | <100 | 0 | <100 | 0 | <100 | 0 | <100 | 0 | <100 | 0 | <100 | 47 | <100 | 0 |
| **3415_V1_C1** | A | 2 | <100 | 0 | <100 | 0 | <100 | 0 | <100 | 0 | <100 | 0 | <100 | 0 | <100 | 0 | <100 | 0 |
| **CAAN5342.A2** | B | 2 | <100 | 0 | – | – | <100 | 0 | <100 | 0 | <100 | 0 | <100 | 0 | <100 | 0 | <100 | 0 |
| **PVO.4** | B | 3 | 113 | 57 | – | – | <100 | 0 | <100 | 0 | <100 | 41 | <100 | 0 | 145 | 58 | <100 | 0 |
| **Q842.D12** | A | 2 | <100 | 43 | – | – | <100 | 0 | <100 | 0 | <100 | 0 | <100 | 0 | <100 | 0 | <100 | 0 |
| **RHPA4259.7** | B | 2 | <100 | 0 | – | – | <100 | 0 | <100 | 0 | <100 | 0 | <100 | 0 | <100 | 0 | <100 | 0 |
| **WITO4160.33** | B | 2 | <100 | 47 | – | – | <100 | 0 | <100 | 0 | <100 | 0 | <100 | 0 | <100 | 0 | <100 | 0 |
| **ZM214M.PL15** | C | 2 | <100 | 0 | – | – | <100 | 0 | <100 | 0 | <100 | 0 | <100 | 0 | <100 | 0 | <100 | 0 |
| **MuLV** | | | <100 | 0 | – | – | <100 | 0 | <100 | 0 | <100 | 0 | <100 | 0 | <100 | 0 | <100 | 0 |
| | | | **M22** | | **M23** | | **M24** | | **M25** | | **M26** | | **M27** | | **M28** | | **M29** | |
| | | | **B4 (week 23)** | | **B4 (week 23)** | | **B4 (week 23)** | | **B4 (week 23)** | | **B4 (week 23)** | | **B4 (week 23)** | | **B4 (week 23)** | | **B4 (week 23)** | |
| **Virus** | **Clade** | **Tier** | **$ID_{50}$** | **% 1:100** | **$ID_{50}$** | **% 1:100** | **$ID_{50}$** | **% 1:100** | **$ID_{50}$** | **% 1:100** | **$ID_{50}$** | **% 1:100** | **$ID_{50}$** | **% 1:100** | **$ID_{50}$** | **% 1:100** | **$ID_{50}$** | **% 1:100** |
| **426c** | C | 2 | <100 | 0 | <100 | 0 | <100 | 0 | <100 | 0 | <100 | 0 | <100 | 0 | <100 | 0 | <100 | 0 |
| **25710** | B | 2 | <100 | 0 | <100 | 0 | <100 | 0 | <100 | 0 | <100 | 0 | <100 | 0 | <100 | 0 | <100 | 0 |
| **CNE8** | AE | 1 | <100 | 0 | <100 | 0 | 100 | 50 | <100 | 0 | 729 | 70 | <100 | 0 | 841 | 61 | 242 | 71 |
| **CNE8 N276A** | AE | 1 | <100 | 0 | <100 | 0 | <100 | 0 | <100 | 0 | <100 | 0 | <100 | 0 | <100 | 0 | <100 | 0 |
| **CNE20** | BC | 2 | <100 | 0 | <100 | 0 | <100 | 0 | <100 | 0 | <100 | 0 | <100 | 0 | <100 | 0 | <100 | 0 |
| **CNE20 N276A** | BC | 2 | <100 | 0 | <100 | 0 | <100 | 0 | <100 | 0 | <100 | 0 | <100 | 0 | <100 | 0 | <100 | 0 |

| | | | | | | | | | | | | | | | | | | |
|---|---|---|---|---|---|---|---|---|---|---|---|---|---|---|---|---|---|---|
| **JRCSF** | B | 2 | <100 | 0 | <100 | 0 | <100 | 0 | <100 | 0 | <100 | 0 | <100 | 0 | <100 | 0 | <100 | 0 |
| **Q23.17** | A | 1 | <100 | 0 | <100 | 0 | <100 | 0 | <100 | 0 | <100 | 0 | <100 | 0 | <100 | 0 | <100 | 0 |
| **YU2** | B | 2 | <100 | 0 | <100 | 0 | <100 | 0 | <100 | 0 | <100 | 0 | <100 | 0 | <100 | 0 | <100 | 0 |
| **BG505 T332N** | A | 2 | <100 | 0 | <100 | 0 | <100 | 0 | <100 | 0 | <100 | 0 | <100 | 0 | <100 | 41 | <100 | 0 |
| **6535.5** | B | 1 | <100 | 0 | <100 | 0 | <100 | 0 | <100 | 0 | <100 | 0 | <100 | 0 | <100 | 0 | <100 | 0 |
| **3415_V1_ C1** | A | 2 | <100 | 0 | <100 | 0 | <100 | 0 | <100 | 0 | <100 | 0 | <100 | 0 | <100 | 0 | <100 | 0 |
| **CAAN534 2.A2** | B | 2 | <100 | 0 | <100 | 0 | <100 | 0 | <100 | 0 | <100 | 0 | <100 | 0 | <100 | 0 | <100 | 0 |
| **PVO.4** | B | 3 | <100 | 0 | <100 | 0 | <100 | 0 | <100 | 0 | <100 | 0 | <100 | 0 | <100 | 43 | <100 | 0 |
| **Q842.D12** | A | 2 | <100 | 0 | <100 | 0 | <100 | 0 | <100 | 0 | <100 | 0 | <100 | 0 | <100 | 0 | <100 | 0 |
| **RHPA425 9.7** | B | 2 | <100 | 0 | <100 | 0 | <100 | 0 | <100 | 0 | <100 | 0 | <100 | 0 | <100 | 0 | <100 | 0 |
| **WITO416 0.33** | B | 2 | <100 | 0 | <100 | 0 | <100 | 0 | <100 | 0 | <100 | 0 | <100 | 0 | <100 | 0 | <100 | 0 |
| **ZM214M. PL15** | C | 2 | <100 | 0 | <100 | 0 | <100 | 0 | <100 | 0 | <100 | 0 | <100 | 0 | <100 | 0 | <100 | 0 |
| **MuLV** | | | <100 | 0 | <100 | 0 | <100 | 0 | <100 | 0 | <100 | 0 | <100 | 0 | <100 | 0 | <100 | 0 |

## Table S6: Oligonucleotides used to generate yeast display gp120 libraries.

| Oligo Name | Fragment | Sequence |
|---|---|---|
| 426c Library 1 For | 1 | GTCTGGAAAGAGGCTAAGACCACACTG |
| 426c Library 1 Rev | 1 | CAGGTTTTTTGATCTGATCACAATCTCTTC |
| 426c Library 1 - 1 For | 2 | GAAGAGATTGTGATCAGATCAAAAAACCTGNNKAACAATGCCAAGATCATTATCGTGC |
| 426c Library 1 - 2 Rev | 2 | ATCTCCACACTCTTATTCAGCTGCACGATAATGATCTTGGCATT |
| 426c Library 1 - 3 For | 2 | AGCTGAATAAGAGTGTGGAGATCGTCTGCACACGACCTAACA |
| 426c Library 1 - 4 Rev | 2 | GCCTGCCGAATATCTCCCCCAGATCCGCTGCCGCCATTGTTAGGTCGTGTGCAGACG |
| 426c Library 1 - 5 For | 2 | GGAGATATTCGGCAGGCTTATTGTAACATCAGTGGCAGAAATTGGTCAGAAGCCGTGAA |
| 426c Library 1 - 6 Rev | 2 | TGGGGGAAGTGCTCTTTCAGCTTTTTCTTGACCTGGTTCACGGCTTCTGACCAATTT |
| 426c Library 1 - 7 For | 2 | AAAGAGCACTTCCCCCATAAGAATATTAGCTTTCAGTCTAGTTCAGGCGGGGAC |
| 426c Library 1 - 8 Rev | 2 | TCGCCTCCGCAGTTGAAGGAGTGTGTGGTGATTTCCAGGTCCCCGCCTGAACTA |
| 426c Library 1 - 9 For | 2 | ACTGCGGAGGCGAGTTCTTTTACTGTAATACATCCGGCCTGTTTAACG |
| 426c Library 1 - 10 Rev | 2 | CCGGCAAGGCAGCATGATTGTGGCATTAGAAATGGTATCGTTAAACAGGCCGGATGTA |
| 426c Library 1 - 11 For | 2 | GCTGCCTTGCCGGATCAAGCAGATTATCAACATGTGGCAGGAA |
| 426c Library 1 - 12 Rev | 2 | TGCCCTTGATGGGTGGTGCATAGATAGCCTTTCCMNNTTCCTGCCACATGTTGATAATC |
| 426c Library 1 - 13 For | 2 | CCACCCATCAAGGGCAATATCACCTGTAAGAGTGACATTACAGGGCTGCTGCTGCTGAGA |
| 426c Library 1 - 14 Rev | 2 | GCCGGAAAATCTCGGTmnnmnnmnnmnnmnnTCCCCCATCTCTCAGCAGCAGCAGCC |
| 426c Library 1 - 15 For | 2 | ACCGAGATTTTCCGGCCTAGCGGAGGAGACATGCGAGATAATTGGCGGTCTGAACTG |
| 426c Library 1 - 16 Rev | 2 | GGATCCCAGAGGCTTGATCTCGACCACCTTATATTTGTACAGTTCAGACCGCCAATTA |
| 426c Library 1 For | 3 | CTGGTGGAGGCGGTAGCGGAGGCGGAGGGTCGGCTAGCGTCTGGAAAGAGGCTAAGACCA |
| 426c Library 1 Rev | 3 | TTACAAGTCCTCTTCAGAAATAAGCTTTTGTTCGGATCCCAGAGGCTTGATCTCGACCAC |
| 426c Library 2 For | 1 | GTCTGGAAAGAGGCTAAGACCACACTG |
| 426c Library 2 Rev | 1 | CAGGTTTTTTGATCTGATCACAATCTCTTC |
| 426c Library 2 - 1 For | 2 | GAAGAGATTGTGATCAGATCAAAAAACCTGNNKAACAATGCCAAGATCATTATCGTGC |
| 426c Library 2 - 2 Rev | 2 | ATCTCCACACTCTTATTCAGCTGCACGATAATGATCTTGGCATT |
| 426c Library 2 - 3 For | 2 | AGCTGAATAAGAGTGTGGAGATCGTCTGCACACGACCTAACA |
| 426c Library 2 - 4 Rev | 2 | GCCTGCCGAATATCTCCCCCAGATCCGCTGCCGCCATTGTTAGGTCGTGTGCAGACG |
| 426c Library 2 - 5 For | 2 | GGAGATATTCGGCAGGCTTATTGTAACATCAGTGGCAGAAATTGGTCAGAAGCCGTGAA |
| 426c Library 2 - 6 Rev | 2 | TGGGGGAAGTGCTCTTTCAGCTTTTTCTTGACCTGGTTCACGGCTTCTGACCAATTT |
| 426c Library 2 - 7 For | 2 | AAAGAGCACTTCCCCCATAAGAATATTAGCTTTCAGTCTAGTTCAGGCGGGGAC |
| 426c Library 2 - 8 Rev | 2 | TCGCCTCCGCAGTTGAAGGAGTGTGTGGTGATTTCCAGGTCCCCGCCTGAACTA |
| 426c Library 2 - 9 For | 2 | ACTGCGGAGGCGAGTTCTTTTACTGTAATACATCCGGCCTGTTTAACG |
| 426c Library 2 - 10 Rev | 2 | CCGGCAAGGCAGCATGATTGTGGCATTAGAAATGGTATCGTTAAACAGGCCGGATGTA |
| 426c Library 2 - 11 For | 2 | GCTGCCTTGCCGGATCAAGCAGATTATCAACATGTGGCAGGAA |
| 426c Library 2 - 12 Rev | 2 | TGCCCTTGATGGGTGGTGCATAGATAGCCTTTCCMNNTTCCTGCCACATGTTGATAATC |
| 426c Library 2 - 13 For | 2 | CCACCCATCAAGGGCAATATCACCTGTAAGAGTGACATTACAGGGCTGCTGCTGCTGAGA |
| 426c Library 2 - 14 Rev | 2 | GCCGGAAAATCTCGGTmnnmnnmnnmnnmnnTCCCCCATCTCTCAGCAGCAGCAGCC |
| 426c Library 2 - 15 For | 2 | ACCGAGATTTTCCGGCCTAGCGGAGGAGACATGCGAGATAATTGGCGGTCTGAACTG |
| 426c Library 2 - 16 Rev | 2 | GGATCCCAGAGGCTTGATCTCGACCACCTTATATTTGTACAGTTCAGACCGCCAATTA |
| 426c Library 2 For | 3 | CTGGTGGAGGCGGTAGCGGAGGCGGAGGGTCGGCTAGCGTCTGGAAAGAGGCTAAGACCA |
| 426c Library 2 Rev | 3 | TTACAAGTCCTCTTCAGAAATAAGCTTTTGTTCGGATCCCAGAGGCTTGATCTCGACCAC |

## Table S7: Flow cytometric reagents

| Reagent | Target species | Antibody clone | Company / Source | Cat.# | RRID |
|---|---|---|---|---|---|
| CD16/32 | mouse | 2.4G2 | BD Biosciences | 553142 | AB_394657 |
| CD4-APCeF780 | mouse | RM4-5 | Thermo Fisher | 47-0042-82 | AB_1272183 |
| CD8a-APCeF780 | mouse | 53-6.7 | Thermo Fisher | 47-0081-82 | AB_1272185 |
| NK1.1-APCeF780 | mouse | PK136 | Thermo Fisher | 47-5941-82 | AB_2735070 |
| F4/80-APCeF780 | mouse | BM8 | Thermo Fisher | 47-4801-82 | AB_2735036 |
| Ly-6G/C (Gr1)-APCeF780 | mouse | RB6-8C5 | Thermo Fisher | 47-5931-82 | AB_1518804 |
| CD11b-APCeF780 | mouse | M1/70 | Thermo Fisher | 47-0112-82 | AB_1603193 |
| CD11c-APCeF780 | mouse | N418 | Thermo Fisher | 47-0114-82 | AB_1548652 |
| CD93-APC | mouse | AA4.1 | Thermo Fisher | 17-5892-82 | AB_469466 |
| TER-119-APCCy0 | mouse | TER-119 | BD Pharmingen | 560509 | AB_1645230 |
| CD95 (FAS)-FITC | mouse | SA367H8 | BioLegend | 152606 | AB_2632901 |
| CD38-AF700 | mouse | 90 | Thermo Fisher | 56-0381-82 | AB_657740 |
| CD45R/B220-BV421 | mouse / human | RA3-6B2 | BD Horizon | 562922 | AB_2737894 |
| CD45R/B220-BV605 | mouse / human | RA3-6B2 | BioLegend | 103244 | AB_2563312 |
| IgD-BV786 | mouse | 11-26c.2a | BD Horizon | 563618 | AB_2738322 |
| CD19-PECy7 | mouse | 6D5 | BioLegend | 115520 | AB_313655 |
| CD2-PE | mouse | RM2-5 | BioLegend | 100108 | AB_2073690 |
| CD23-PE | mouse | B3B4 | BioLegend | 101607 | AB_312832 |
| Ig light chain lambda-APC | mouse | RML-42 | BioLegend | 407306 | AB_961363 |
| Ig light chain kappa-BV421 | mouse | 187.1 | BD Horizon | 562888 | AB_2737867 |
| CD21/CD35 | mouse | 7G6 | BD Horizon | 562756 | AB_2737772 |
| IgM Fab-FITC | mouse | polyclonal | Jackson Immunoresearch | 115-097-020 | AB_2338618 |
| Zombie NIR | N/A* | N/A | BioLegend | 423105 | N/A |
| Streptavidin-PE | N/A | N/A | BD Pharmingen | 554061 | AB_10053328 |
| Streptavidin-AF647 | N/A | N/A | BioLegend | 405237 | N/A |
| Streptavidin-PECy7 | N/A | N/A | BioLegend | 405206 | N/A |

| | | | | | |
|---|---|---|---|---|---|
| RC1-biotin | N/A | N/A | in house | N/A | N/A |
| CNE8 N276A-biotin | N/A | N/A | in house | N/A | N/A |
| 426c degly2 D279N-biotin | N/A | N/A | in house | N/A | N/A |
| 426c degly2 D279N CD4bs-KO -biotin | N/A | N/A | in house | N/A | N/A |
| Human Fc Block | human | N/A | BD Horizon | 564220 | AB_2869554 |
| Ig light chain lambda-APC | human | MHL38 | BioLegend | 316610 | AB_493629 |
| CD19-PECy7 | human | SJ25C1 | BioLegend | 363012 | AB_2564203 |
| IgM-FITC | human | MHM88 | BioLegend | 314506 | AB_493009 |
| Ig light chain kappa-BV421 | human | MHK-49 | BioLegend | 316518 | AB_2561581 |

*N/A not applicable

## Table S8: Single cell antibody cloning reaction conditions.

| PCR1 IgH | Primer sequence |
|---|---|
| HH_1FL (forward, leader) | CCATGGGATGGTCATGTATCA |
| HH_1RG (reverse, IgG) | GGACAGGGATCCAGAGTTCC |
| HH_1RM (reverse, IgM) | CCCATGGCCACCAGATTCTT |

| PCR1 IgK | Primer sequence |
|---|---|
| HH_1FL (forward, leader) | CCATGGGATGGTCATGTATCA |
| HH_1RK (reverse, IgK) | GACTGAGGCACCTCCAGATG |

**PCR1 mastermix**

| Reagent | Volume/plate (µL) | Concentration |
|---|---|---|
| nuclease free water | 3328 | |
| 10x buffer | 384 | 1x |
| dNTP (25 mM) | 48 | 0.3 mM |
| 5' forward Primer (50 µM) | HC 15; LC 19 | HC 0.25 µM; LC 0.25 µM |
| 3' reverse Primer (50 µM) | HC 23 (IgG/IgM 1:1); LC 19 | HC 0.30 µM; LC 0.25 µM |
| HotStar DNA Polymerase (5 U/µL) | 42 | 0.055 U/µL |
| total | 3840 | |

| PCR2 IgH | Primer sequence |
|---|---|
| HH_2FL (forward, leader) | GTAGCAACTGCAACCGGTGTACATTCT |
| HH_2RG (reverse, IgG) | GCTCAGGGAARTAGCCCTTGAC |
| HH_2RM (reverse, IgM) | AGGGGGAAGACATTTGGGAAGGAC |

| PCR2 IgK | Primer sequence |
|---|---|
| HH_2FL (forward, leader) | GTAGCAACTGCAACCGGTGTACATTCT |
| HH_2RK (reverse, IgK) | AACTGCTCACTGGATGGTGG |

**PCR2 mastermix**

| Reagent | Volume/plate (µL) | Concentration |
|---|---|---|
| nuclease free water | 2536 | |
| loading buffer* | 800 | |
| 10x buffer | 384 | 1x |
| dNTP (25 mM) | 48 | 0.3 mM |
| 5' forward Primer (50 µM) | HC 12; LC 15 | HC 0.16 µM; LC 0.2 µM |
| 3' reverse Primer (50 µM) | HC 18 (IgG/IgM 1:1); LC 15 | HC 0.23 µM; LC 0.2 µM |
| HotStar DNA Polymerase (5 U/µL) | 42 | 0.055 U/µL |
| total | 3840 | |

*loading buffer: 40% (w/v) sucrose in nuclease free water with cresol red added to dark red color.
